# AccessPD as a next generation registry to accelerate Parkinson’s disease research

**DOI:** 10.1038/s41531-024-00651-z

**Published:** 2024-03-19

**Authors:** Yun-Hsuan Chang, Maria Teresa Periñan, Matt Wilson, Alastair J. Noyce

**Affiliations:** 1 UMEDEOR LTD, 8 Warner Yard, London, EC1R 5EY UK; 2 Unidad de Trastornos del Movimiento, Servicio de Neurología y Neurofisiología Clínica, Instituto de Biomedicina de Sevilla, Hospital Universitario Virgen del Rocío/Consejo Superior de Investigaciones Científicas (CSIC)/Universidad de Sevilla, 41013 Seville, Spain; 3 Centre for Preventive Neurology, Wolfson Institute of Population Health, Faculty of Medicine and Dentistry, Queen Mary University of London, London, UK

**Keywords:** Parkinson's disease, Medical research

## Abstract

Recruitment is a major rate-limiting factor in Parkinson’s disease (PD) research. AccessPD is a unique platform that aims to create a registry of more than 2000 PD patients and a rich database of PD-relevant information. Potential participants are identified using electronic health records (EHRs) in primary care. They are contacted via text message with an individualized link to the study portal. Electronic patient-reported outcomes (ePRO) are collected via online questionnaires and integrated with existing EHR. 200 participants were recruited within the first 6 months, of which 191 answered the follow-up questionnaire. Here, to showcase the potential of AccessPD, we described the most common diagnoses before and after PD diagnosis, the most commonly prescribed drugs, and identified participants who could benefit from device-aided therapies using consensus criteria. AccessPD shows its unique ability to link different data sources for patient stratification in longitudinal studies and recruitment into clinical trials.

## Introduction

Parkinson’s disease (PD) is a complex neurological condition with a broad range of clinical symptoms, which is influenced by both genetic and environmental factors^1^. Management of PD calls for individualized pharmacological and non-pharmacological treatment. More research is needed to understand PD etiology, sub-types, progression rates, and phenotype-genotype correlations in order to optimize management^2,3^. Two needs for PD research are: (1) efficient ways to improve rate of participant recruitment, and (2) access to high-quality data from a diverse and representative sample of the population with PD. Inequity in access to research opportunities due to socioeconomic or geographical factors continues to bias our understanding of PD causation, manifestations, treatment adherence and response, and limits the generalizability of findings^4^.

The widespread adoption of electronic health record (EHR) systems and increasing use of EHR data for secondary use (e.g., research) presented an opportunity to redesign a next-generation disease registry that is dynamic, interoperable, and has the ability to effectively connect and combine data from different sources^5^. This differs from traditional registries which require collaboration between large medical centers and active involvement of the clinicians to identify and collate cases^6^.

Here, we describe AccessPD, a registry that aims to accelerate PD research by supporting participant enrollment and facilitating the collection of longitudinally linked data for patient stratification. The system utilizes EHR data collected at the point of care at primary care practices across England to identify potential participants with a confirmed diagnosis of PD. Once a patient is contacted and consented into the registry, electronic patient-reported outcomes (ePRO), which are key indicators for disease progression and management, are collected via regular online questionnaires and integrated with existing EHR data, together with genetic or biomarker data that are obtained from home testing. Approved researchers can take advantage of the large, growing database and conduct novel studies to further our understanding of the disease. Partners wishing to validate devices or enroll participants to clinical trials can use AccessPD to recruit highly stratified patients. The remote nature of the registry ensures that participation is accessible to a more diverse population with PD than is typically seen in research studies.

This report summarises the recruitment of the first 200 patients to the AccessPD registry, and showcases the type of data that can be curated from re-engagement with questionnaires and from the EHR. We then show how this information can be used to stratify participants for precision research opportunities, using a specific case study to identify candidates with motor fluctuations to enroll in trials of device-aided therapies.

## Results

### Initial engagement and baseline questionnaire

We identified a total of 1676 (0.27%) PD patients out of 628,610 unique patients registered with the first 51 participating general practitioner (GP) practices, as of March 1, 2023. Of the 1676 patients contacted for initial engagement, 404 (24.1%) responded by clicking on the link included in the text invitation and 232 (13.8%) participants completed all the consent questions. 200 consented and agreed to take part in AccessPD. The 200th patient with PD was recruited into AccessPD 182 days after the launch of the study.

We observed a balanced representation of both genders among the consented participants and all the PD patients invited to participate. Regarding age, there was an overrepresentation of AccessPD patients in the range between 70 and 79 years when compared to the invited group (see Supplementary Table 1).

Of the first 200 participants, 43.0% were female and 57.0% were male (Table 1). The most commonly reported symptoms were tremor (78.0%), muscle stiffness (64.5%), slowness of movement (58.5%), fatigue (60.0%), sleep disorder (49.0%), and gait problems (45.0%). A family history of PD was reported in 19.0%. Two (1%) participants had undergone deep brain stimulation.

**Table 1:** Participant demographic data and self-reported symptoms for the first 200 participants

AccessPD as a next generation registry to accelerate Parkinson’s disease research		
Characteristics		Participants (*n* = 200)
Average age (years) at diagnosis (95% CI)		65.6 (64.2, 67.0)
Average years since diagnosis (95% CI)		5.2 (4.5, 5.9)
Average age (years) at enrollment (95% CI)		70.8 (69.5, 72.1)
Age at enrollment (years)	<40	1 (0.5%)
	40–49	5 (2.5%)
	50–59	13 (6.5%)
	60–69	56 (28.0%)
	70–79	97 (48.5%)
	>80	28 (14.0%)
Sex at birth, *n* (%)		
	Female	86 (43.0%)
	Male	114 (57.0%)
Self-reported ethnicity, *n* (%)		
	White (including English, Welsh, Scottish, Northern Irish or Irish and any other White background)	189 (94.5%)
	Non-white (including Asian, black, mixed, other, and prefer not to say)	11 (5.5%)
Index of Multiple Deprivation^a^ of the postcode areas where the participants live		
	Index of Multiple Deprivation Decile (from most deprived to least deprived)	Number of postcode areas, *n* (%)
	1–2	24 (12.4%)
	3–4	35 (18.1%)
	5–6	17 (8.8%)
	7–8	44 (22.8%)
	9–10	73 (37.8%)
Most common current symptoms, *n* (%)		
	Tremor	156 (78.0%)
	Muscle stiffness	129 (64.5%)
	Fatigue	120 (60.0%)
	Slowness of movement	117 (58.5%)
	Problem sleeping	98 (49.0%)
	Problem walking	90 (45.0%)
Family History of PD, *n* (%)		
	Yes	38 (19.0%)
	No	162 (81.0%)
Impact of PD on movement, *n* (%)		
	No impact of motor symptoms	27 (13.5%)
	Symptoms affect one side of the body	73 (36.5%)
	Symptoms affect both sides of the body	23 (11.5%)
	Symptoms affect both sides of the body and walking/balance is impaired	55 (27.5%)
	Assistance in daily activities needed	22 (11.0%)
History of Deep Brain Stimulation, *n* (%)		
	Yes	2 (1.0%)
	No	198 (99.0%)

^a^In the English Indices of Deprivation, a lower index value indicates a higher degree of deprivation in the area. United Kingdom Government. English Indices of Deprivation 2019. (2019). https://www.gov.uk/government/statistics/english-indices-of-deprivation-2019.

The average age at the time of recruitment was 70.8 years. The youngest participant was 39-year-old and the oldest was 90-year-old. 18 (9.0%) participants were diagnosed before the age of 50. With an average number of years since diagnosis of 5.2 years, the participant with the longest history of PD was diagnosed 31 years ago, and 15% of participants were diagnosed within the past 24 months (Supplementary Table 2).

50% of the participants reported they had either no PD motor symptoms or their symptoms were confined to only one side of the body. 39% of participants reported that both sides of their bodies were affected by PD or they struggled with walking and balance. 11% of participants need assistance with activities of daily living.

Analysis of the IMD scores showed that >30% of participants lived in the lowest two quintiles of deprivation in England (IMD 1–4). When asked about their ethnicity, the majority (94.5%) of the participants identified as white, with the remainder disclosing that they were Asian, mixed, black, or preferred not to answer.

### First engagement questionnaire

A total of 191 registered participants answered the first follow-up questionnaire, 55 of them required assistance from a nurse via telecommunication to enter their responses. The survey results showed that 84.3% of the participants were comfortable with using mobile technology (laptop, tablet, computer, or mobile phone) as the primary way for receiving communication. 78.5% of the participants have not participated in any form of PD-related research. Of those who had research experience in the past, the most common type was questionnaire-based research (52.2%), followed by clinical research that required trial site visits (21.3%) and research involving a medical device or testing kit (14.8%).

**Fig. 1:**
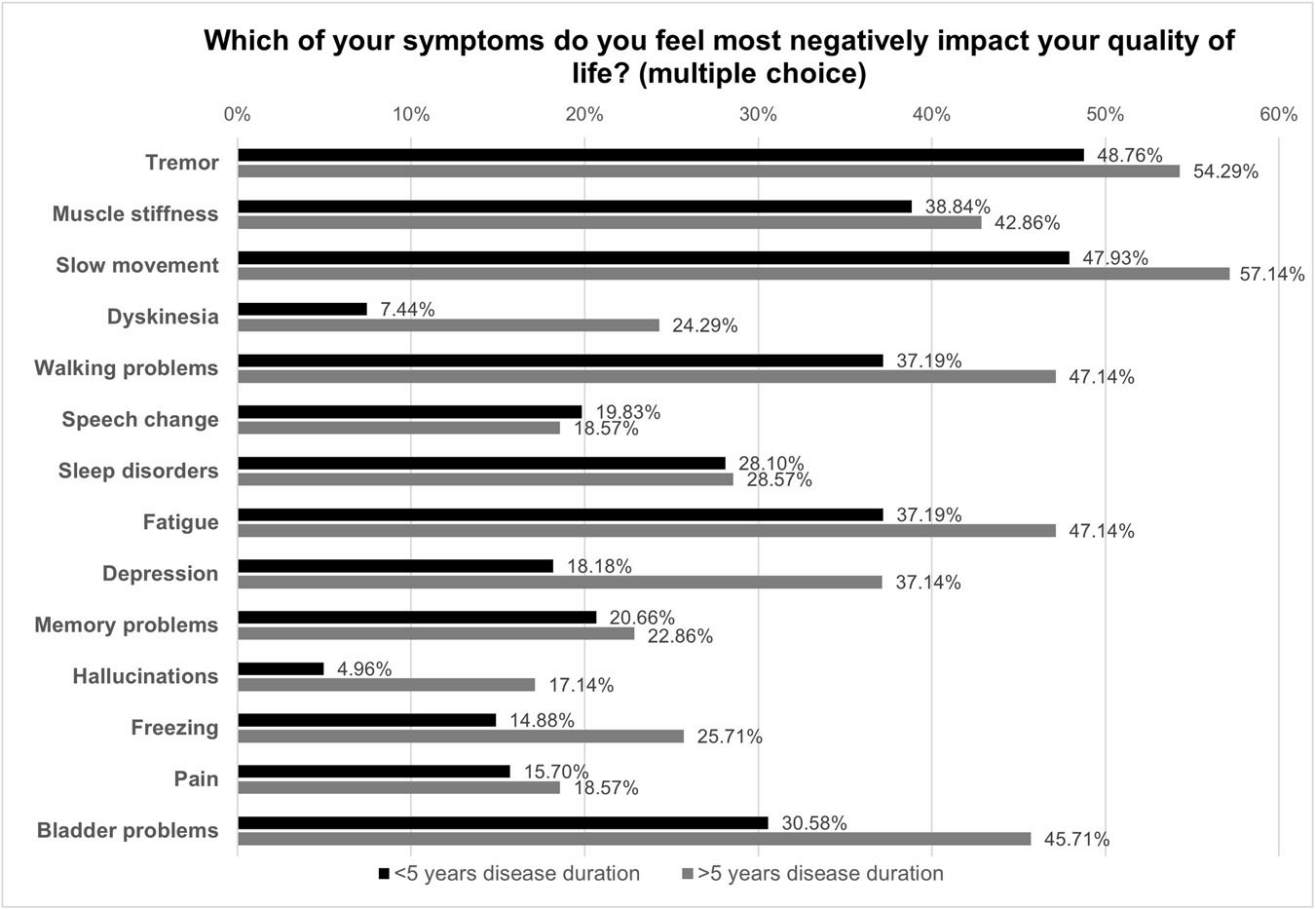
Impact of Symptoms on Quality of Life. Bar charts depicting the impact of symptoms on quality of life among participants diagnosed more than 5 years ago versus those diagnosed less than 5 years ago.

When asked about the symptoms that most negatively impacted the quality of life, the most common answers among those diagnosed within 5 years were tremor (48.8%), slow movement or loss of dexterity (47.9%), muscle stiffness (38.8%), walking problems (37.2%), and fatigue (37.2%). Meanwhile, individuals with a disease history of more than 5 years most commonly reported slow movement (57.1%), tremor (54.3%), walking problems (47.1%), fatigue (47.1%), and bladder problems (45.7%) (see Fig. 1). It is noteworthy that participants with a disease duration exceeding 5 years were three times more likely to report dyskinesia compared to those with shorter disease duration (24.3% vs. 7.3%). Likewise, the prevalence of depression was twice as high among participants with a longer disease duration (37.1% vs. 18.2%).

Among all participants, fatigue (22.0%), tremor (20.9%), walking problems (20.4%), bladder or bowel function disorders (20.4%), and slowness of movement (19.4%) were identified as the most common symptoms least controlled by their current medication. In general, 64.9% of participants said they were satisfied with their current therapy and management of PD.

In all, 36.8% of participants reported suffering from ‘off’ periods. The remaining participants had either not noticed any ‘off’ periods or were unsure about this, and 6 (3%) responded that they were not yet on PD medication. We asked if participants were aware of device-aided therapy for PD. Only 6 out of 191 (3%) participants were aware of deep brain stimulation. One participant knew about apomorphine infusion or subcutaneous apomorphine, and one had heard of levodopa-carbidopa intestinal gel.

One of the 191 participants who responded to the follow-up questionnaire reported having multiple system atrophy-parkinsonian type (MSA-P) and was excluded from further analysis.

### Analysis of ePROs and EHR data

83.9% of participants had a coded diagnosis of PD that first appeared within one year of the self-reported date of diagnosis (Table 2). In most instances (80.2%) the self-reported date appeared earlier than the first EHR record of PD.

**Table 2:** Comparison between self-reported date of diagnosis with the first appearance of PD diagnosis in the EHR

AccessPD as a next generation registry to accelerate Parkinson’s disease research	
Lapse between self-reported date of diagnosis and first appearance of PD diagnosis in EHR	No. of patients, *n* (%)
Within 1 year	161 (83.9)
Between 1–2 years	14 (7.3)
Between 2–3 years	4 (2.1)
Between 3–4 years	5 (2.6)
Between 4–5 years	2 (1.0)
More than 5 years	6 (3.1)
**Self-reported date earlier than first appearance of PD diagnosis in EHR**	154 (80.2)
**First appearance of PD diagnosis in EHR earlier than self-reported date of diagnosis**	37 (19.3)

Patients without a coded diagnosis of PD were excluded from this analysis. To compare self-reported date of diagnosis in the baseline questionnaire with EHR record of PD diagnosis, observations and medication records of the first 200 participants were extracted. 7 participants were identified using their prescription records and had no coded diagnosis of PD in EHR. 1 participant was excluded due to their MSA-P diagnosis. The total number of participants included in the analysis was 192.

Table 3 summarises the most common visit-associated diagnoses extracted from EHR records of the 190 participants, before and after their PD diagnosis. Musculoskeletal conditions, hypertensive disorder, depression and anxiety, urinary tract infection, skin conditions, hemorrhoids or constipation, and respiratory tract infection are among the most frequent reasons for a GP consultation both before and after the diagnosis. 14.2% of patients had a record indicating diabetes after PD diagnosis (versus 5.3% before the diagnosis).

**Table 3:** Most common co-existing visit-associated diagnoses of the participants, before and after their PD diagnosis

AccessPD as a next generation registry to accelerate Parkinson’s disease research					
	Before diagnosis of PD		After diagnosis of PD		
	Diagnosis/symptom	No. of patients, *n* (%)		Diagnosis/symptom	No. of patients, *n* (%)
1	Joint pain & musculoskeletal disorders	75 (39.5)	1	Joint pain & musculoskeletal disorders	68 (35.8)
2	Skin lesions, dermatitis, & keratosis	57 (30.0)	2	Hypertensive disorder	61 (32.1)
3	Hypertensive disorder	32 (16.8)	3	Depression & anxiety	32 (16.8)
4	Headache/migraine	28 (14.7)	4	Urinary tract infection	31 (16.3)
=4	Depression & anxiety	28 (14.7)	5	Skin lesions, dermatitis & keratosis	30 (15.8)
6	Hemorrhoids or constipation	25 (13.2)	6	Diabetes	27 (14.2)
=6	Abdominal hernia	25 (13.2)	7	Hemorrhoids or constipation	20 (10.5)
8	Respiratory tract infection	24 (12.6)	8	Asthma	19 (10.0)
=8	Dyspepsia/indigestion/reflux	24 (12.6)	9	Respiratory tract infection	19 (10.0)
10	Urinary tract infection	21 (11.1)	10	Rheumatoid arthritis	12 (6.3)

The equal sign in the ranking column indicates the same code frequency.

A similar analysis was performed to list the most commonly prescribed drugs in the EHR starting from 2021, one year prior to enrollment. Drugs of the same class, such as statins, were grouped together. Other than levodopa and COVID-19 vaccines, statins, proton-pump-inhibitors, macrogol, and selective serotonin reuptake inhibitors (SSRIs) were among the most frequently prescribed drugs (Table 4).

**Table 4:** The 10 most commonly prescribed drugs recorded in the EHR since 2021 (a year before the start of the enrollment)

AccessPD as a next generation registry to accelerate Parkinson’s disease research	
Name of medication	Number of patients, *n* (%)
Levodopa (co-beneldopa and co-careldopa)	142 (74.7)
COVID-Vaccine	131 (69.0)
Omeprazole/Esomeprazole	43 (22.6)
Statin (Rosuvastatin, Atorvastatin, Simvastatin, Pravastatin)	43 (22.6)
Macrogol	38 (20.0)
SSRI (Escitalopram, Citalopram, Sertraline, Fluoxetine)	31 (16.3)
Rasagiline	31 (16.3)
Amoxicillin/Penicillin	26 (13.7)
Paracetamol	26 (13.7)
Doxycycline	25 (13.2)

In reference to the 5-2-1 criteria, we identified potential candidates for device-aided therapies. It is crucial to emphasize that our application of these criteria was conducted loosely, serving as an initial screening method. The characteristics of identified candidates should be thoroughly confirmed in a clinical setting before any changes to management, whether for routine care or research-related activities, are made. Based on EHR medication records, 18 (9.5%) participants were prescribed levodopa 5 times daily, while 56 (29%) participants reported dyskinesia in the baseline questionnaire and 70 (37%) participants answered yes to wearing-off symptoms in the follow-up questionnaire. This meant that a total of 95 (50%) participants were either on levodopa five times daily *or* reported wearing-off *or* dyskinesia. Of these, 58 (61.1%) participants had been prescribed adjunctive PD medications from one of the three drug groups: Dopamine Agonists (DA), Monoamine Oxidase Inhibitors (MAOi), or Catechol-O-Methyltransferase (COMT) inhibitors. Within this subgroup of 58 participants, 43 (74.1%) were prescribed at least one drug from one of the three groups, 12 (20.1%) have been prescribed drugs from two of the groups, whilst three participants (5.2%) had been trialed on drugs from all three classes of adjunctive treatment.

## Discussion

Here, we describe the creation and launch of the AccessPD registry: a next-generation platform to accelerate PD research. The principal goal of AccessPD is to accelerate progress by providing access to opportunities for patients, access to patients for researchers, and access to data for the research community. The ability to re-engage AccessPD participants rapidly and create highly stratified groups of patients using EHR information and self-reported data, makes this accelerated pathway for research tangible. Integration of DNA and biomarker collection over the next couple of years and further growth of the registry are planned.

The successful recruitment of the first 200 participants to the registry within 6 months of launch demonstrates the capability of EHR data in supporting targeted trial recruitment. On average, 7.7 participants were recruited into AccessPD per week, a figure that is higher than the weekly average of 4.9 participants reported in a review of three similar decentralized studies conducted by Myers et al.^7^ Interestingly, 62.5% of the first 200 participants were above 70 years of age and ~80% had never been involved in research. This not only reflects the higher prevalence of PD among older patients but also suggests that older age is not a barrier to participating in a digital disease registry such as AccessPD. Although the IMD scores, calculated using postcodes of the participants, serve only as a proxy for measuring relative deprivation, they indicate that participants were recruited from a wide range of socioeconomic backgrounds^8^.

One of the limitations of traditional registries is the lack of ability to keep pace with changing requirements and the cumbersome nature of re-engaging and re-consenting participants at scale for collection of additional data points or dissemination of the latest PD-related study information^9^. AccessPD’s strength in efficiently re-engaging participants in this regard is evidenced by the speed and rate of response to our first follow-up questionnaire where 95.5% of participants submitted their answers within 2 weeks. This differentiates AccessPD from other research databases that solely support population health research and lack the ability to re-engage participants. One notable observation from the follow-up questionnaire was the threefold higher prevalence of dyskinesia among individuals with an extended disease duration. This aligns with expectations, as dyskinesia often emerges as a side effect of prolonged levodopa treatment^10^. This example underscores that the data collected from our participants are indicative of common symptoms and typical disease progression among PD patients.

28.8% of participants required help from a nurse to complete the first questionnaire. The intention is to assist them in ensuring accurate setup from the outset, with the nurse’s involvement primarily focused on onboarding. Subsequently, participants should be self-sufficient, requiring minimal assistance for future follow-up data collections. Completely automating the process risks excluding individuals less familiar with technology or from lower socioeconomic backgrounds. By taking this approach, we ensure a more inclusive and supportive experience for all participants.

We envisage a huge range of possibilities for AccessPD, including: (1) researchers being able to engage participants with remote collections of data and samples (e.g., questionnaires, biospecimens), (2) rapid testing and validation of devices and software designed for patients with PD, (3) rapid recruitment to investigator-led and/or commercial clinical trials. We conducted four separate analyses to demonstrate both the integration of data and its validity. We observed reassuring results with respect to EHR date of diagnosis and self-reported diagnosis (>90% concordant within 0–2 years) and expected results for concurrent diagnoses and prescriptions held in the EHR.

We then used a case study focused on identifying those who might be suitable for either new research studies or who might be considered for a change in clinical management. We used consensus criteria (5-2-1 criteria) to identify 58 potentially eligible AccessPD participants^11^. Of note, only 3% of AccessPD participants were previously aware of device-aided therapies. The ability to stratify patients precisely according to the inclusion and exclusion criteria of a given research opportunity offers unparalleled efficiency in recruitment from the perspective of the research sponsors and participants. As mentioned above, the 5-2-1 criteria were loosely applied in the analysis. However, we could potentially introduce wearable devices to characterize the “off” period and enhance its specificity.

The AccessPD model disrupts the traditional, inefficient, and costly model in which those that develop devices or drugs, go one-by-one to specialists in secondary care to find patients for their trials. By shifting the focus to primary care, stratifying patients, and engaging directly with those patients, much of the bias and exclusivity of research to date is eroded. This is demonstrated by the inclusion of older participants in AccessPD and the spread of participants across different multiple deprivation indices, factors that often lead to exclusion from previous research opportunities due to age or financial burden^12^.

The study is not without its limitations. To maximize the sensitivity of our search algorithm, we utilized both diagnosis and drug codes for the identification of potential PD patients. A participant is then asked to confirm their PD diagnosis during the registration. The problem with this approach is two-fold: patients who are approached based on their prescription data alone, but who do not have PD, may perceive the invitation to the registry as intrusive. Furthermore, the medication algorithm does not identify early-stage patients with PD who are not on medication, potentially skewing the distribution of participants toward later-stage patients. Conversely, individuals aged over 80 and those reliant on daily assistance exhibited lower participation rates, thereby contributing to selection bias and constraining the applicability of our findings to the broader population. Despite our efforts to facilitate participation through the nursing team, individuals in care homes, as well as those with advanced PD and dementia, were unable to participate.

Using EHR data for secondary research comes with intrinsic limitations such as data inconsistency, incompleteness, and inaccuracy, as they are “by-products” of routinely collected data. Physicians might use different codes to represent the same disease, or code a symptom rather than a diagnosis during consultation, thus making it challenging to determine the most frequent concurrent diagnoses. Nevertheless, EHR data is valuable in providing researchers a general idea of co-morbidities which could be used to form the basis of new hypotheses. By linking ePROs and EHR data, AccessPD provides a unique way to cross-reference and validate information.

As mentioned above, the current distribution of participants across strata of deprivation is highly encouraging. However, the proportion of non-white participants in the registry remains relatively low. This could be attributed to factors such as language barriers and limited diversity among the background population of primary care providers involved in the pilot project, concentrated in Southeast and Northwest England. Improving diversity in AccessPD remains a priority. In this regard, we are implementing measures, such as upgrading our system to facilitate multilingual engagements and actively exploring partnership with GPs in more diverse neighborhoods to further promote inclusivity. Moreover, recognizing the overall low consent rate (200 out of 1676) observed during the pilot, we have made adjustments, including experimenting with various invitation messages and modifying language used in patient-facing content, to enhance participation.

Finally, we acknowledge that certain PD symptoms affecting cognitive function, such as hallucinations and memory impairment, might be challenging for participants to self-report. As their disease progresses, participants may require additional support in responding to engagements. We will explore the possibility of involving caregivers and family members in questionnaires that concern cognitive functions in the future.

In conclusion, the decentralized design of AccessPD enables study opportunities to be offered to participants that are often overlooked and aims to erode biases in participant selection and improve generalisability of results. More work will be done to understand the low uptake of AccessPD among ethnic minority groups and develop enhancement strategies.

Our current effort is focused on increasing the number of participants in AccessPD, collecting DNA and information from wearable devices, and seeking partners who wish to recruit study participants to drug trials or device validation studies. We also welcome researchers to access the existing data to tackle important research questions.

## Methods

### Engagement, recruitment, and high-level design of AccessPD

**Fig. 2:**
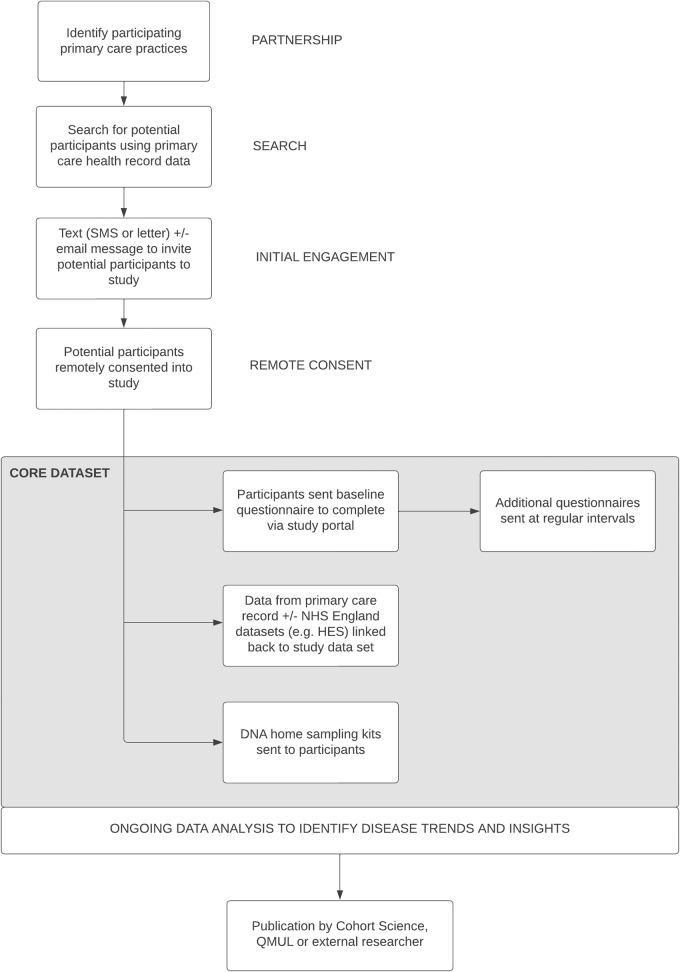
Design of AccessPD. Through partnerships with primary care providers, uMed acts on behalf of the GPs to identify potential candidates for AccessPD. Interested participants are engaged and consented remotely. NHS National Health Services, HES Hospital Episode Statistics, QMUL Queen Mary University of London.

AccessPD recruits patients via a digital platform powered by uMedeor LTD (uMed), a life sciences research organization that acts as a data processor for a network of primary care GPs across England. uMed enables GPs to engage in research studies by automating the identification of potential study participants, a time-consuming step for the clinical staff, and facilitating participant engagement on behalf of the GPs. uMed’s system routinely incorporates EHR data from GP surgeries, subsequently subjecting it to comparison against a set of predefined search criteria to pinpoint potential participants with a PD diagnosis. This is a dynamic process that adds new patients to the pool of invitees. The registry is run and maintained by Cohort Science, a clinical research organization, in collaboration with researchers from Queen Mary University of London (QMUL). The study protocol was approved by East Midlands - Derby Research Ethics Committee in May 2022. Figure 2 demonstrates the high-level design of the data ecosystem.

Patients with a coded diagnosis of PD – which is predominantly represented by the Systematised Nomenclature of Medicine Clinical Terms (SNOMED-CT) code 49049000 in primary care EHR – were identified as potential participants (additional diagnostic codes are listed in Supplementary Table 3). As PD is primarily managed in secondary or tertiary care in England, diagnosis records might be missing in primary care EHR systems. To mitigate the challenge of undercoding in primary care data, patients with a prescription of one or more anti-parkinsonian drugs were also identified as a subcohort of potential participants (Supplementary Table 4). Participants had to be above the age of 18, with capacity to give consent and have regular access to the internet or telecommunications.

With approval from participating GPs to engage these patients, uMed sends out a letter to inform them of the study opportunity, followed by a text message (either SMS or Email message) with an individualized link to the study portal. Once eligibility is established, participants give their consent to join the registry by going through a series of interactive questions. Alternatively, those less familiar with remote studies can request assistance from a study nurse who will go through the consent questions on the telephone with them and record answers in the database.

Incorporation of hospital episodes and electronic prescribing data from secondary and tertiary care is planned in the long-term design. In addition, the database will include genetic data and biomarker results from home testing kits. Approved researchers, affiliated or not with Cohort Science and QMUL, can access this resource via a secure web portal and conduct PD-relevant research. All participants will be informed of any use of their data in research and have the opportunity to opt out, should they wish to.

While maintaining data security and privacy, participants in the registry will be regularly informed of new study outcomes and opportunities via newsletters and re-consented for changes in study or privacy requirements, if necessary.

33 GP practices across England were involved in the project at launch in September 2022. The number of participating practices increased to 51 by the time the first 200 participants were recruited. All practices use either EMIS Health (90.2%) or SystemOne (9.8%), two of the main primary care EHR systems in the UK.

### Demographic data at baseline

Data such as age at enrollment, average years since PD diagnosis, ethnicity, and gender were extracted from questionnaire-responses of the first 200 participants. Using postcodes stored in EHR records, and the 2019 English deprivation data published by the Department for Levelling Up, Housing and Communities in the UK, we extracted the indices of multiple deprivation (IMD) for the 193 postcode areas where the participants reside. IMD is a score based on a number of socio-economic domains such as income, employment, education, and health to represent relative deprivation of each small area in the UK^13^. A lower index value indicates a higher degree of deprivation in the area. Descriptive data are presented as the mean and 95% confidence interval (CI) for parametric data. Categorical data are presented as proportions.

### Collecting ePROs and linking data

Consented participants received a baseline questionnaire immediately after enrollment, collecting information on their demographics and current symptoms. The assessment of symptoms and medication will occur at regular intervals of every 6 months. Validated questionnaires, on the other hand, will be distributed at varying intervals, specifically every 6 to 18 months, depending on the intention of the questionnaire. The first follow-up questionnaire on symptoms and medication consisting of 15 multiple-choice questions (see Supplementary Table 5) was sent out in March 2023 to the first 197 participants.

To create a linked database, the primary care EHR data of the consented participants were incorporated into the registry to provide a longitudinal view of the medical history. Information was extracted separately from EMIS and SystemOne. The EMIS Data Extraction Service was used to acquire data from practices using the EMIS Health system, whilst a proprietary tool developed by uMed was used to obtain data from collaborating SystemOne practices.

### Data linkage and processing

To demonstrate the ability of AccessPD to provide clinical insights, we conducted several analyses that utilized both ePROs and EHR data.

We compared the self-reported date of diagnosis with the diagnosis records of the first 200 participants extracted from primary care EHR. Inaccuracy in recall or delay in entering PD diagnosis into primary care EHR could lead to minor discrepancies between recalled and recorded date of diagnosis. As PD is a chronic disease and some participants only remember the closest month of diagnosis, we considered a difference of less than 12 months between the two dates as acceptable. Participants identified by their prescription records alone were excluded from this comparison because the first appearance of a PD medication prescription has limited relevance in determining the date of PD diagnosis if no treatment was commenced immediately.

By analyzing EHR observation records, we created a list of the most commonly used diagnostic codes among the participants before and after their diagnosis of PD. This analysis serves to provide a general insight into EHR data of the participants by looking at the codes that were most frequently applied during their encounters with their primary care providers.

To create a dataset for observation records, we first extracted the EMIS (carerecord_observation) and SystemOne (srcode) records separately and then joined the two tables using SNOMED-CT identifiers. The records were divided into two different datasets that represent all observation codes prior to and after the self-reported year of diagnosis for each individual patient.

Since the observation records contain more than just diagnoses and symptoms and certain diagnoses are represented by multiple codes (e.g., both “asthma” and “annual review of asthma” indicate the presence of an asthma diagnosis), the list of the most commonly used codes was reviewed by a clinician to keep only the relevant codes. Codes that represent administrative information or laboratory tests were discarded and not included in the ranking (see Supplementary Table 6 for codes discarded and Supplementary Table 7 for codes used in querying each symptom/diagnosis).

As with the observation records, we joined the prescription records of EMIS (Prescribing_DrugRecord) and SystemOne (SRPrimaryCareMedication) by dm+d codes that are common to both systems. Only records from 2021 onward were taken into account. After removing duplicate drug prescriptions for each individual, we listed out the 10 most commonly prescribed drugs among the participants at the point of enrollment.

Participants who had either been prescribed levodopa five times daily *or* had self-reported periods of ‘off’ time *or* dyskinesia were identified based on EHR records and answers in the questionnaires, respectively. This approach roughly aligns with the 5-2-1 screening criteria, which is ≥5 doses of oral levodopa/day, or “off” symptoms for ≥2 hours/day, or ≥1 hour of dyskinesia/day, that are used to identify patients with advanced PD who may benefit from device-aided therapies^11,14^. We then looked at EHR prescriptions to see if they had previously been treated with dopamine agonists, MAO-B inhibitors or COMT inhibitors. We used this case study to show how AccessPD could be used for a specific indication or to stratify patients for precision opportunities, such as identifying patients with complex PD who might be suitable for device-aided therapies.

### Reporting summary

Further information on research design is available in the Nature Research Reporting Summary linked to this article.

### Supplementary information

Supplemental Material

Reporting summary

## Data Availability

The datasets generated and the underlying code for this study are not publicly available but may be made available to qualified researchers upon request which will be evaluated based on ethical, legal, and privacy considerations by the corresponding author.
