# Supplementary material for: AccessPD as a next generation registry to accelerate Parkinson’s disease research
Source: NPJ Parkinsons Dis. 2024 Mar 19;10:66. doi: 10.1038/s41531-024-00651-z (PMC10950855; doi:10.1038/s41531-024-00651-z)
Supplement: Supplementary file 1 — Supplemental Material [file 41531_2024_651_MOESM1_ESM.pdf]

Supplementary Material: AccessPD - a ‘next generation’ registry to accelerate Parkinson’s disease research

Supplementary Table 1. Age and sex distribution of individuals invited to participate (All identified PD patients in the 51 practices) versus those who consented to participate (AccessPD participants)..2

Supplementary Table 2. Breakdown of participants by disease duration................................2

Supplementary Table 3. Diagnostic codes for Parkinson’s disease..2

Supplementary Table 4. Drug codes for anti-parkinsonian drugs including levodopa, dopamine agonists, monoamine oxidase-B (MAO-B) inhibitors, Catechol O-Methyltransferase (COMT) inhibitors.. 3

Supplementary Table 5. Template for the first follow-up questionnaire...................................7

Supplementary Table 6. Codes discarded when analysing the most common co-exisiting diagnoses before and after the confirmation of Parkinson’s disease. These codes are discarded as they do not represent or indicate the presence of a medical diagnosis............10

Supplementary Table 7. Codes used to search for each of the top 10 most common co-existing diagnoses before and after the confirmation of Parkinson’s disease..................33

Supplementary Table 1. Age and sex distribution of individuals invited to participate (All identified PD patients in the 51 practices) versus those who consented to participate (AccessPD participants).

AccessPD participants			All identified PD patients in the 51 practices		
**Age**	<40	0.5%	**Age**	<40	0.6%
	40-49	2.5%		40-49	1.8%
	50-59	6.5%		50-59	9.0%
	60-69	28.0%		60-69	23.1%
	70-79	48.5%		70-79	38.5%
	>80	14.0%		>80	26.9%
**Average age**		70.8	**Average age**		73.7
**Male**		57.0%	**Male**		59.1%
**Female**		43.0%	**Female**		40.9%

Supplementary Table 2. Breakdown of participants by disease duration.

Disease duration	% Participants
< 5 years	56%
5 - 9 years	26%
10 - 15 years	12.5%
> 15 years	5.5%

Supplementary Table 3. Diagnostic codes for Parkinson's disease.
The following SNOMED-CT codes indicate the presence of a PD diagnosis and were used in the search for PD patients. Some patients might have multiple of these codes in their EHR records. The number of patients identified using each code only gives a rough estimate of their frequency of use. They do not represent the actual number of patients engaged because the database searched contained only anonymised data which means patients who had deceased or were deregistered from GP practices in the uMed's network were not excluded. Furthermore, as of March 2023, only 51 practices within uMed's network participated in the recruitment.

SNOMED ID	Description	Number of patients with this code
49049000	Parkinson's disease	4951
907151000000108	Seen by Parkinson's disease service	957
425390006	Dementia associated with Parkinson's Disease	452
862081000000106	Referral to Parkinson's service	337

924261000000104	Referral to community Parkinson's disease clinical nurse specialist	270
879471000000102	Referral to community Parkinson's service	58
515841000000104	History of Parkinson's disease	44
718685006	Orthostatic hypotension co-occurrent and due to Parkinson's disease	9
101421000119107	Dementia due to Parkinson's disease	8
341551000000108	Cerebral degeneration in Parkinson's disease	2
715345007	Young onset Parkinson disease	1

Supplementary Table 4. Drug codes for anti-parkinsonian drugs including levodopa, dopamine agonists, monoamine oxidase-B (MAO-B) inhibitors, Catechol O-Methyltransferase (COMT) inhibitors.

SNOMED Code	Description
323107008	Product containing precisely benserazide 12.5 milligram and levodopa 50 milligram/1 each conventional release oral capsule
323108003	Product containing precisely benserazide 25 milligram and levodopa 100 milligram/1 each conventional release oral capsule
323109006	Product containing precisely benserazide 50 milligram and levodopa 200 milligram/1 each conventional release oral capsule
323110001	Co-beneldopa 12.5mg/50mg dispersible tablet
323112009	Co-beneldopa 25mg/100mg dispersible tablet
323119000	Product containing precisely carbidopa 12.5 milligram and levodopa 50 milligram/1 each conventional release oral tablet
323208008	Product containing precisely pramipexole dihydrochloride 88 microgram/1 each conventional release oral tablet
323209000	Product containing precisely pramipexole dihydrochloride 180 microgram/1 each conventional release oral tablet
323210005	Product containing precisely pramipexole dihydrochloride 700 microgram/1 each conventional release oral tablet
377268007	Product containing precisely carbidopa 10 milligram and levodopa 100 milligram/1 each conventional release oral tablet
377269004	Product containing precisely carbidopa 25 milligram and levodopa 100 milligram/1 each conventional release oral tablet
377270003	Product containing precisely carbidopa 25 milligram and levodopa 250 milligram/1 each conventional release oral tablet
323184004	Product containing precisely ropinirole (as ropinirole hydrochloride) 1 milligram/1 each conventional release oral tablet
13639711000001102	Ropinirole 2mg modified-release tablets
3910921000001100	Co-careldopa 50mg/200mg modified-release tablets

11178911000001104	Caramet 25mg/100mg CR tablets (Teva UK Ltd)
5299611000001105	Stalevo 100mg/25mg/200mg tablets (Orion Pharma (UK) Ltd)
15440811000001105	Stalevo 75mg/18.75mg/200mg tablets (Orion Pharma (UK) Ltd)
5302211000001106	Stalevo 150mg/37.5mg/200mg tablets (Orion Pharma (UK) Ltd)
15440311000001101	Stalevo 125mg/31.25mg/200mg tablets (Orion Pharma (UK) Ltd)
13644811000001105	Stalevo 200mg/50mg/200mg tablets (Orion Pharma (UK) Ltd)
5298311000001106	Stalevo 50mg/12.5mg/200mg tablets (Orion Pharma (UK) Ltd)
19701511000001106	Stalevo 175mg/43.75mg/200mg tablets (Orion Pharma (UK) Ltd)
39734911000001107	Co-careldopa 25mg/250mg tablets
39734611000001101	Co-careldopa 10mg/100mg tablets
39734811000001102	Co-careldopa 25mg/100mg tablets
39734711000001105	Co-careldopa 12.5mg/50mg tablets
14054611000001101	Co-careldopa 25mg/100mg/5ml oral solution
11815111000001106	Co-careldopa 25mg/100mg/5ml oral suspension
8427211000001103	Co-careldopa 12.5mg/50mg/5ml oral suspension
8427611000001101	Co-careldopa 6.25mg/25mg/5ml oral suspension
39109011000001105	Co-careldopa 25mg/100mg modified-release tablets
391092110000011006	Co-careldopa 50mg/200mg modified-release tablets
10161711000001105	Co-careldopa 5mg/20mg/1ml intestinal gel 100ml cassette
27911000001105	Madopar CR 125 capsules (Roche Products Ltd)
379211000001105	Madopar 250 capsules (Roche Products Ltd)
129911000001109	Madopar 125 capsules (Roche Products Ltd)
73911000001102	Madopar 62.5 capsules (Roche Products Ltd)
570711000001108	Madopar 125 dispersible tablets (Roche Products Ltd)
192211000001104	Madopar 62.5 dispersible tablets (Roche Products Ltd)
562411000001105	Sinemet 25mg/250mg tablets (Organon Pharma (UK) Ltd)
790711000001101	Sinemet 10mg/100mg tablets (Organon Pharma (UK) Ltd)
236411000001107	Sinemet 12.5mg/50mg tablets (Organon Pharma (UK) Ltd)
826111000001108	Sinemet CR 50mg/200mg tablets (Organon Pharma (UK) Ltd)
210411000001104	Sinemet Plus 25mg/100mg tablets (Organon Pharma (UK) Ltd)
242011000001103	Half Sinemet CR 25mg/100mg tablets (Organon Pharma (UK) Ltd)
39707211000001101	Co-beneldopa 25mg/100mg dispersible tablets sugar free
39706911000001107	Co-beneldopa 12.5mg/50mg dispersible tablets sugar free
10161911000001107	Duodopa intestinal gel cassette 100ml (Solvay Healthcare Ltd) |
3661711000001106	Mirapexin 700microgram tablets (Boehringer Ingelheim Ltd)
13127011000001109	Mirapexin 350microgram tablets (Boehringer Ingelheim Ltd)
3663011000001105	Mirapexin 180microgram tablets (Boehringer Ingelheim Ltd)

3663411000001101	Mirapexin 88microgram tablets (Boehringer Ingelheim Ltd)
16162611000001100	Mirapexin 2.1mg modified-release tablets (Boehringer Ingelheim Ltd)
16162911000001106	Mirapexin 3.15mg modified-release tablets (Boehringer Ingelheim Ltd)
18042311000001102	Mirapexin 1.57mg modified-release tablets (Boehringer Ingelheim Ltd)
18042611000001107	Mirapexin 2.62mg modified-release tablets (Boehringer Ingelheim Ltd)
16162011000001107	Mirapexin 0.52mg modified-release tablets (Boehringer Ingelheim Ltd)
323186002	Product containing precisely ropinirole (as ropinirole hydrochloride) 5 milligram/1 each conventional release oral tablet
323185003	Product containing precisely ropinirole (as ropinirole hydrochloride) 2 milligram/1 each conventional release oral tablet
323183005	Product containing precisely ropinirole (as ropinirole hydrochloride) 250 microgram/1 each conventional release oral tablet
13639911000001100	Ropinirole 8mg modified-release tablets
13639811000001105	Ropinirole 4mg modified-release tablets
13639711000001102	Ropinirole 2mg modified-release tablets
29184311000001107	Ropinirole 3mg modified-release tablets
29184411000001100	Ropinirole 6mg modified-release tablets
379611000001107	ReQuip 2mg tablets (GlaxoSmithKline)
481511000001109	ReQuip 250microgram tablets (GlaxoSmithKline)
923211000001100	ReQuip 5mg tablets (GlaxoSmithKline)
268111000001107	ReQuip 1mg tablets (GlaxoSmithKline)
3891211000001106	ReQuip tablets starter pack (GlaxoSmithKline)
3889511000001109	ReQuip tablets follow on pack (GlaxoSmithKline)
13628411000001102	ReQuip XL 4mg tablets (GlaxoSmithKline)
13628111000001107	ReQuip XL 2mg tablets (GlaxoSmithKline)
13628711000001108	ReQuip XL 8mg tablets (GlaxoSmithKline)
35935911000001108	Rotigotine 4mg/24hours transdermal patches
10142111000001106	Rotigotine 8mg/24hours patches
35936011000001100	Rotigotine 6mg/24hours transdermal patches
16683311000001101	Rotigotine 1mg/24hours transdermal patches
16665711000001104	Rotigotine 3mg/24hours transdermal patches
35935811000001103	Rotigotine 2mg/24hours transdermal patches
10129911000001102	Neupro transdermal patches starter pack (Schwarz Pharma Ltd)
10127311000001108	Neupro 2mg/24hours transdermal patches (Schwarz Pharma Ltd)
10128911000001105	Neupro 6mg/24hours transdermal patches (Schwarz Pharma Ltd)
10128311000001109	Neupro 4mg/24hours transdermal patches (Schwarz Pharma Ltd)
10129411000001105	Neupro 8mg/24hours transdermal patches (Schwarz Pharma Ltd)
16669211000001109	Neupro 1mg/24hours transdermal patches (UCB Pharma Ltd)

16664911000001101	Neupro 3mg/24hours transdermal patches (UCB Pharma Ltd)
5322411000001101	Levodopa 150mg / Carbidopa 37.5mg / Entacapone 200mg tablets
15457511000001109	Levodopa 75mg / Carbidopa 18.75mg / Entacapone 200mg tablets
5322511000001102	Levodopa 50mg / Carbidopa 12.5mg / Entacapone 200mg tablets
13648111000001109	Levodopa 200mg / Carbidopa 50mg / Entacapone 200mg tablets
5322311000001108	Levodopa 100mg / Carbidopa 25mg / Entacapone 200mg tablets
15457411000001105	Levodopa 125mg / Carbidopa 31.25mg / Entacapone 200mg tablets
19744111000001107	Levodopa 175mg / Carbidopa 43.75mg / Entacapone 200mg tablets
418125001	Rasagiline 1mg tablets
9362611000001103	Azilect 1mg tablets (Teva UK Ltd)
323158005	Selegiline 5mg tablets
323157000	Selegiline 10mg tablets
35938811000001107	Selegiline 10mg/5ml oral solution
323155008	Selegiline 1.25mg oral lyophilisates sugar free
32696511000001102	Safinamide 50mg tablets
32696411000001101	Safinamide 100mg tablets
323202009	Entacapone 200mg tablets
8487111000001103	Entacapone 200mg/5ml oral suspension
5322411000001101	Levodopa 150mg / Carbidopa 37.5mg / Entacapone 200mg tablets
15457511000001109	Levodopa 75mg / Carbidopa 18.75mg / Entacapone 200mg tablets
5322511000001102	Levodopa 50mg / Carbidopa 12.5mg / Entacapone 200mg tablets
13648111000001109	Levodopa 200mg / Carbidopa 50mg / Entacapone 200mg tablets
5322311000001108	Levodopa 100mg / Carbidopa 25mg / Entacapone 200mg tablets
15457411000001105	Levodopa 125mg / Carbidopa 31.25mg / Entacapone 200mg tablets
19744111000001107	Levodopa 175mg / Carbidopa 43.75mg / Entacapone 200mg tablets
375993004	Tolcapone 100mg tablets
33495211000001103	Opicapone 50mg capsules
33494911000001108	Ongentys 50mg capsules (BIAL Pharma UK Ltd)
323133000	Amantadine 100mg capsules
35899611000001103	Amantadine 50mg/5ml oral solution sugar free
35902411000001102	Apomorphine 30mg/3ml solution for injection pre-filled disposable devices
35902611000001104	Apomorphine 50mg/5ml solution for injection ampoules
35902311000001109	Apomorphine 20mg/2ml solution for injection ampoules
134614006	Apomorphine 2mg sublingual tablets sugar free
134615007	Apomorphine 3mg sublingual tablets sugar free
35902511000001103	Apomorphine 50mg/10ml solution for infusion pre-filled syringes
36441811000001103	Apomorphine 30mg/3ml solution for injection cartridges

36441211000001104	Apomorphine 100mg/20ml solution for infusion vials

Supplementary Table 5. Template for the first follow-up questionnaire

Question
Q1: Do you prefer to answer the questionnaires that we send to you on your mobile, tablet, laptop or speaking over the phone ?
Mobile
Tablet
Laptop
Over the phone
Letter
Other (please specify)
Q2: Have you been offered the chance to participate in Parkinson's research before or is Access-PD the first opportunity?
ACCESS-PD is the first
1-5 studies
>5 studies
other
Q3: If you have participated in Parkinson's research before, what type of research was this (you can select multiple options)?
Questionnaire-based
Involved a medical device or testing kit
Clinical trial completed at home only
Clinical trial completed involving travel (to a hospital or GP surgery)
I've never participated in Parkinson's research before AccessPD
Q4: Are you satisfied with your current medication / therapy for Parkinson's?
Yes
No
Other (please specify)
Q5: What would be your preferred method of taking your Parkinson's medication?
Pill once a day
Pill multiple times per day
Multiple pills multiple times per day
Skin patches
Injections
Infusions
Other (Please specify)
Q6: Which of your symptoms do you feel most negatively impacts your quality of life (you can select

multiple options)?
Tremor
Muscle Stiffness
Slowness of movement or loss of dexterity
Dyskinesia (uncontrolled, involuntary movement)
Walking problems (abnormal gait, difficulty walking, falls)
Speech change
Sleep disorders
Fatigue
Depression or anxiety
Memory problems
Hallucinations
Freezing (suddenly not be able to move forward for several seconds or minutes, feeling like your lower body is stuck)
Pain
Bladder or bowel problems
Other (please specify)
Q7: Which of your symptoms do you feel most negatively impacts your ability to carry out daily activities (you can select multiple options)?
Tremor
Muscle Stiffness
Slowness of movement or loss of dexterity
Dyskinesia (uncontrolled, involuntary movement)
Walking problems (abnormal gait, difficulty walking, falls)
Speech change
Sleep disorders
Fatigue
Depression or anxiety
Memory problems
Hallucinations
Freezing (suddenly not be able to move forward for several seconds or minutes, feeling like your lower body is stuck)
Pain
Bladder or bowel problems
Other
Q8: Which symptoms do you feel are best treated by your current medication (you can select multiple options)?
Tremor

Muscle Stiffness
Slowness of movement or loss of dexterity
Dyskinesia (uncontrolled, involuntary movement)
Walking problems (abnormal gait, difficulty walking, falls)
Speech change
Sleep disorders
Fatigue
Depression or anxiety
Memory problems
Hallucinations
Freezing (suddenly not be able to move forward for several seconds or minutes, feeling like your lower body is stuck)
Pain
Bladder or bowel problems
Other (please specify)
Q9: Which symptoms do you feel are least helped by your current medication?
Tremor
Muscle Stiffness
Slowness of movement or loss of dexterity
Dyskinesia (uncontrolled, involuntary movement)
Walking problems (abnormal gait, difficulty walking, falls)
Speech change
Sleep disorders
Fatigue
Depression or anxiety
Memory problems
Hallucinations
Freezing (suddenly not be able to move forward for several seconds or minutes, feeling like your lower body is stuck)
Pain
Bladder or bowel problems
Other (please specify)
Q10: Do you suffer from on/off periods (medications wear off gradually or abruptly, resulting in the return of Parkinson’s symptoms)?
Yes
No
Other

Q11: If you answered ‘Yes’, does your medication do a good job of managing the number of on/off periods?
Yes
No
I do not suffer from on/off periods
Other (please specify)
Q12: Do you do anything or take anything in addition to your medication to manage on/off periods?
Yes (please specify)
No
I do not suffer from On/Off periods
Other (please specify)
Q13: Have you switched medication that you take for Parkinson’s in the past and if so, what reasons did you have for switching?
Medication did not improve symptoms (please specify the main symptom that medication did not help)
Side effects
Cost
Injection site reactions (if injected medication)
Other (please specify)
Q14: Have you ever been made aware of or been offered a device aided therapy by your physician?
Yes - deep brain stimulation
Yes - apomorphine infusion / sub-cutaneous apomorphine
Yes - levodopa-carbidopa intestinal gel
No
Not sure
Other
Q15: Do you have any agitators or specific tasks that cause your symptoms to increase? An example could be that flossing your teeth causes your tremor to increase.
Yes - Please specify
No

Supplementary Table 6. Codes discarded when analysing the most common co-existing diagnoses before and after the confirmation of Parkinson’s disease. These codes are discarded as they do not represent or indicate the presence of a medical diagnosis.

**Before the confirmation of PD diagnosis**	
**SNOMED codes**	**Description**
184229000	Notes summary on computer

965831000000103	Implied consent for core Summary Care Record dataset upload
773011000000101	Patient allocated named accountable general practitioner
313334002	Blood sample taken
908481000000105	Informing patient of named accountable general practitioner
266919005	Never smoked tobacco
182836005	Telephone encounter
279991000000102	Administrative procedure
185317003	Medication review
14734007	Medication review done
314530002	Medication review with patient
88551000000109	Patient review
763288003	Result
394617004	British or mixed British - ethnic category 2001 census
183049006	Ex-smoker
92391000000108	Did not attend - no reason
11816003	Awaiting clinical code migration to EMIS Web
8517006	Preferred method of contact: unknown
270426007	SMS (short message service) text message sent to patient
15728710000061005	O/E - pulse rhythm regular
185210004	Failed encounter - message left on answer machine
95324001	SMS text message sent to patient
185324002	Patient mobile telephone number
183093006	Current non-smoker
279039007	Had a chat to patient
185340004	Discharge summary
428481002	Medication requested
160618006	Main spoken language English
373942005	Informed consent for procedure
171071003	Choice and booking enhanced services administration
315570003	Bowel cancer screening programme
182771004	FBC - full blood count
222711000000102	Exercise grading
29857009	NHS Health Check completed
286901000000107	Failed encounter
523221000000100	Letter from specialist
30989003	Letter encounter
49727002	Seen in hospital casualty

45326000	Telephone triage encounter
59621000	Advice on foreign travel
160628002	Administration
308720009	Letter from consultant
225323000	Patient's condition improved
401267002	Dietary advice
416118004	GFR (glomerular filtration rate) calculated by abbreviated Modification of Diet in Renal Disease Study Group calculation
275693003	Marital state unknown
398838000	Seen in GP's surgery
1020291000000100	Seen in dermatology clinic
160504008	Enjoys moderate exercise
185202000	Seen in orthopaedic clinic
270422009	Lifestyle education
313204009	Patient-initiated diet
172630005	Patient given advice
160632008	Serum lipids level
160660005	Mammography normal
304507003	Cervical smear - negative
185252009	Thyroid function test
46825001	Health education - exercise
767357000	White British
168749009	Bowel cancer screening programme faecal occult blood test normal
185228002	Enjoys light exercise
1005661000000100	Patient informed - test result
167261002	Urine glucose test negative
165355002	Systolic arterial pressure
269958004	Referral for further care
315236000	Depression screening using questions
160631001	Medication review of medical notes
20135006	Diastolic arterial pressure
10168510000001005	Sampling of cervix for Papanicolaou smear done
183444007	General practice physical activity questionnaire physical activity index: active
60621009	Body mass index
93311000000106	Smoking cessation advice
200971000000100	Seen in urology clinic
366241000000106	Education about alcohol consumption

81680005	Date records held from
72313002	General practice physical activity questionnaire physical activity index: moderately active
161891005	Letter encounter from patient
162415008	White British - ethnic category 2001 census
162031009	Liver function test
165333005	Urine protein test negative
50417007	Seen in cardiac clinic
281078001	MED3 - doctor's statement
997531000000108	Letter received
25064002	Advice about treatment given
185220009	Standing height
494131000000105	Serum alanine aminotransferase level
314940005	Serum albumin level
167273002	Standard ECG
397803000	Syringing ear to remove wax
270424005	Seen in ophthalmology clinic
164847006	Advice to patient - subject
171024009	Seen in neurology clinic
1016851000000100	Blood test requested
248333004	Serum potassium level
1321000000100	Nucleated red blood cell count
185222001	Body weight
715931000000100	Serum urea level
1000821000000100	Serum sodium level
198291000000105	Serum creatinine level
21522001	Ca cervix - screening done
1000751000000100	NHS Health Check invitation first letter
1000731000000100	Renal profile
1000951000000100	Serum TSH (thyroid stimulating hormone) level
27113001	Serum high density lipoprotein cholesterol level
1000651000000100	Pulse rate
182531007	Seen in ENT clinic
185230000	Serum cholesterol level
1022461000000100	Serum alkaline phosphatase level
268543007	Dressing of wound
1000661000000100	Seen in radiology department
163133003	Letter from outside agency

185223006	Serum total bilirubin level
160617001	Stopped smoking
19231000000106	Haematocrit
314471005	Lymphocyte count
160573003	Haemoglobin estimation
185181002	Abdomen examined - NAD
1005671000000100	Patient offered choice of provider
78564009	MCH - Mean corpuscular haemoglobin
997591000000109	Blood sample -> Lab NOS
359748005	Red blood cell count
1022791000000100	Urine dipstick test
1000621000000100	Total white cell count
1005681000000100	Platelet count
1022511000000100	Seen in general surgery clinic
286261000000101	Fax sent to:
359748005	Patient's condition the same
183545006	MCV - Mean corpuscular volume
1010671000000100	Neutrophil count
1022291000000100	Forms - miscellaneous
1022581000000100	Diet health education
24671000000101	Telephone call to a patient
1006761000000100	Plasma glucose level
400010006	Monocyte count
201101007	Bone profile
168731009	Bowel cancer screening programme: faecal occult blood result
408947007	Eosinophil count
1941000000108	Serum triglycerides level
82078001	Declined consent for short message service text messaging
1022551000000100	Electronic record notes summary verified
699237001	Letter sent to patient
14221000000108	Serum cholesterol/high density lipoprotein ratio
1007881000000100	Screening - general
70153002	Hypertension monitoring
1022591000000100	No known allergy
1022431000000100	Failed encounter - no answer when rang back
1022651000000100	Discharge summary report
12063002	Serum low density lipoprotein cholesterol level

1022491000000100	X-ray report received
519211000000103	Basophil count
25581000000101	Urinalysis = no abnormality
249274008	Serum calcium level
80313002	Lloyd George record received
267102003	Telephone consultation
185276001	ECG
62315008	Seen in physiotherapy department
167221003	Diet good
1015681000000100	Urine nitrite negative
2051000000104	Has authorisation for medication under PSD (patient specific direction)
1005691000000100	Advice given
716186003	Med3 certificate issued to patient
1022191000000100	Urine ketone test negative
1022561000000100	Urea and electrolytes level
14760008	Lloyd George culled and summarised
11441004	Refer to physiotherapist
368481000000103	MCHC - Mean corpuscular haemoglobin concentration
272039006	Health education - alcohol
185337004	Standard chest X-ray
15188001	Letter encounter to patient
49218002	Haemoglobin A1c level - International Federation of Clinical Chemistry and Laboratory Medicine standardised
275944005	Letter sent to consultant
935051000000108	Minor surgery done - injection
167287002	Urine sample sent to Lab
23056005	Urine blood test = negative
160639004	Reassurance given
164226003	Aerobic exercise 3+ times/week
165339009	Mole of skin
314138001	Seen in gastroenterology clinic
14679004	High fibre diet
386472008	Brief intervention for physical activity completed
999791000000106	ESR - erythrocyte sedimentation rate
84089009	Seasonal influenza vaccination
47933007	Inhaler technique - good
171324002	Wax in ear
1000971000000100	Review of medication

183039008	Electrocardiographic monitoring
1746291000006100	Ex-moderate cigarette smoker (10-19/day)
195967001	Test result to patient by telephone
185245007	Health education offered
367391008	Hormone replacement therapy
15805002	GPPAQ hours in last week spent walking - 3 hours or more
202855006	New patient screening done
271739002	Blood withdrawal for testing
822851000000102	Patient registration data verified
375031000000109	New patient screening
170625000	Occupations
168336001	Transfer-degraded record entry
49049000	Aerobic exercise 2 times/week
266717002	Moderate drinker - 3-6u/day
1022261000000100	Seen in accident and emergency department
165357005	Minor surgery done + claimable
24079001	Serum adjusted calcium concentration
300471006	GPPAQ hrs in last wk spent in physical exercise - 3hrs or more
60862001	Electrocardiographic procedure
713019007	General surgical referral
1746371000006100	GPPAQ usual level of walking pace - steady
414941008	Discussion about disorder
195742007	Cardiac disease monitoring
171175005	Consultation
196411000000103	Liquid based cervical cytology screening
183665006	Indirect encounter
174184006	Medication given
16991000000107	Seen by optician
38341003	GPPAQ not in employment
366121000000108	ECG normal
25721000000107	Alcohol intake
703938007	O/E - blood pressure reading
183542009	Spirometry screening
254701007	Discharged from hospital
394700004	Scanned document
182833002	O/E - heart sounds normal
77176002	General practice physical activity questionnaire physical activity index: inactive

1746271000006100	Follow-up arranged
185316007	Raised blood pressure
185719002	Lifestyle advice regarding hypertension
10601006	Patient advised about exercise
164854000	No response to bowel cancer screening programme invitation
223471001	Chaperone offered
1104081000000100	Laboratory test requested
443402002	Acute kidney injury warning stage
24761000000103	Refer to practice nurse
183616001	Referral letter
29303009	Message given to patient
163074001	Patient's condition worsened
171255006	Seen by practice phlebotomist
217082002	Seen in minor injuries department
68566005	Enjoys heavy exercise
183544005	Provision of chaperone refused
163020007	Spirometry
173422009	Discussion about therapy
276074009	Choose and book electronic referral letter sent
11429006	Inhaler technique observed
373251000000108	Did not attend
401118009	Diagnostic colonoscopy
183073003	QRISK2 cardiovascular disease 10 year risk score
268509003	Failed encounter - message left with household member
49650001	Seen by consultant
417036008	E-mail sent to patient
182832007	Drug compliance checked
993381000000106	Urine culture
160633003	Medication review without patient
239872002	Urine leukocyte test = negative
92531000000104	Ex-light cigarette smoker (1-9/day)
25611000000107	Removal of suture from skin
86094006	Patient registration
419603000	Clinical history and observation findings
266922007	Mail administration procedure
271299001	Dermatological referral
74506000	Failed encounter NOS

127783003	Ambulatory blood pressure recording
763380007	Serum ferritin level
281399006	Serum electrolytes level
184047000	Report administration
1.00064E+15	Ethnic category not stated - 2001 census
183096003	Self-help advice leaflet given
307780009	12 lead ECG
17741008	Application of dressing
391156007	Current smoker
74016001	No breathlessness
41446000	Resp. system examined - NAD
394717006	Low fat diet education
167217005	Drug compliance good
718087004	Laboratory test observable
513861000000102	Framingham coronary heart disease 10 year risk score
113091000000109	Plasma fasting glucose level
176178006	History / symptoms
185293001	ENT referral
183523005	Histology laboratory test
1000641000000100	GPPAQ hours in last week spent gardening/DIY - none
170614009	Liver function tests - general
3895009	Breast neoplasm screening normal
450759008	Nursing procedure
183518005	Letter sent to outside agency
401270003	Urine test for glucose
408373006	Trivial drinker - <1u/day
609558009	Non-smoker
139394000	A&E report
160237006	On examination - right dorsalis pedis pulse present
223485006	Health education - breast examination
713541000000102	Seen in vascular clinic
1003181000000100	Alcohol consumption
185190009	Blood test due
75088002	Urine examination
161938003	Examination / signs
160593006	MED5 issued to patient
993411000000108	Lloyd George and problem summary

185438005	On examination - left dorsalis pedis pulse present
275926002	Aerobic exercise 1 time/week
1371000000101	Serum folate level
78809005	Medication review done by pharmacist
992831000000108	Consent given for blood test
160637002	Prescription collected by pharmacy
125605004	Seen in audiology clinic
44054006	Minor surgery done - excision
9632001	Email sent to patient
9826008	O/E - gait normal
184103008	Clinical letter
182884001	Travel destination
363746003	Serum total protein
823691000000103	Communication from:
417662000	eConsultation via online application
719329004	DNA hospital appointment
24561000000109	Vascular disease risk assessment
939511000000101	Administration note
23919004	Registered for online access to local practice
268926000	Bereavement
177860007	Motor vehicle traffic accident
314481009	Alcohol intake within recommended sensible limits
183061003	Screening procedure
397881000	Patient medical record envelope received from family practitioner committee
10509002	Light drinker - 1-2u/day
77477000	Incoming mail NOS
162356005	Diagnostic cystoscopy
161894002	Consent given to share patient data with specified third party
16791000000109	Breast neoplasm screening
195647007	Over the counter aspirin therapy
315217005	Dissent from disclosure of personal confidential data by Health and Social Care Information Centre
235595009	Dissent from secondary use of general practitioner patient identifiable data
34014006	PSA (prostate-specific antigen) level
1031081000000100	Seen in walk in centre
1746451000006100	Brief intervention for excessive alcohol consumption completed

163120009	Chest clear
198436008	Histopathology test
165349007	Normal histology findings
8392000	Test request : Full Blood Count
170638008	Last menstrual period -1st day
268547008	Diagnostic arthroscopy of knee joint
163684001	Gastroscopy
17293009	Letter invite to screening
313148005	Medication changed
440401008	Smoking cessation education
364712009	Patient reassurance
261000000103	Medication management
37351000000107	Seen by specialist physician
394726009	Cervical smear - 1st call
170635006	Seen in hospital out-pat.
183521007	Intramuscular injection
1030791000000100	Influenza vaccination invitation first letter sent
267032009	Gynaecological referral
1746411000006100	Urine protein test = +
343121000000108	Ex-heavy cigarette smoker (20-39/day)
185248009	Tobacco smoking consumption
705055006	Computer summary updated
308021002	ENT examination - NAD
40701008	10g monofilament sensation R foot normal
30037006	Adult health examination
306635009	Referral to G.P.
287721000000104	Seen in out of hours centre
522261000000101	Low cholesterol diet education
203351000000102	Drugs not issued
183830008	Minor surgery done - cautery
47382004	Prescription collected by patient
281794004	On examination - right posterior tibial pulse present
182918009	Fall - accidental
394670005	INR - international normalised ratio
90834002	BP screening - first recall
17369002	Referral letter sent
105542008	Choose and book referral

161920001	New registration check done and claimable
267432004	Total cholesterol:HDL (high density lipoprotein) ratio
24420007	Cardiovascular disease risk assessment done
167297006	Adult screening
1022481000000100	Orthopaedic referral
310500000	SMS text message received from patient
302415002	GPPAQ hours in last week spent cycling - none
270224008	Married
53726008	Serum free T4 level
14361000000109	Incoming mail
35489007	Consent given for communication by text messaging
171302002	Microbiology
1016971000000100	MSU sent for C/S
**After the confirmation of PD diagnosis**	
**SNOMED codes**	**Description**
279991000000102	SMS (short message service) text message sent to patient
428481002	Patient mobile telephone number
185230000	Seen in neurology clinic
313334002	Blood sample taken
185317003	Telephone encounter
182836005	Telephone consultation
25721000000107	Never smoked tobacco
266919005	Template entry - EHR composition type
182771004	Medication review
386472008	Informed consent for procedure
88551000000109	Medication review with patient
788007007	General practice service
314530002	Medication review done
162999005	O/E - pulse rhythm regular
310080006	Pharmacy service
270426007	Did not attend - no reason
184103008	Weight monitoring
270426007	Patient telephone number
307818003	Immunisation course to maintain protection against SARS-CoV-2 (severe acute respiratory syndrome coronavirus 2)
1362591000000100	Medication requested
182888003	Administrative procedure

14734007	Ex-smoker
8517006	Failed encounter - message left on answer machine
185324002	Failed encounter
185340004	Administration note
37351000000107	Preferred method of contact: unknown
822701000000109	Has authorisation for medication under PSD (patient specific direction)
1990681000006100	eConsultation via online application
1068881000000100	Enhanced review indicated before granting access to own health record
185210004	Choice and booking enhanced services administration
1364731000000100	Systolic arterial pressure
72313002	Diastolic arterial pressure
222711000000102	GFR (glomerular filtration rate) calculated by abbreviated Modification of Diet in Renal Disease Study Group calculation
1091811000000100	Letter from specialist
1020291000000100	Telephone triage encounter
270425006	Serum potassium level
401267002	Serum urea level
1000651000000100	Informing patient of named accountable general practitioner
1000731000000100	Serum sodium level
1000661000000100	Serum creatinine level
908481000000105	FBC - full blood count
1000951000000100	Body weight
1022441000000100	Patient allocated named accountable general practitioner
11816003	Serum alanine aminotransferase level
27113001	Haemoglobin estimation
965831000000103	Serum alkaline phosphatase level
1018251000000100	Letter from consultant
1022431000000100	Urea and electrolytes level
1000971000000100	Platelet count
1000621000000100	MCV - Mean corpuscular volume
183049006	Red blood cell count
275693003	Patient given advice
394617004	Monocyte count
1022581000000100	Neutrophil count
1000821000000100	Seen in hospital casualty
1022651000000100	Result
1022571000000100	Lymphocyte count
1022451000000100	Haematocrit

1022491000000100	Total white cell count
1022551000000100	Signposting
975131000000104	Eosinophil count
699237001	Serum albumin level
1022591000000100	Basophil count
1022561000000100	Bowel cancer screening programme faecal occult blood test normal
1022291000000100	Discharge summary
1022541000000100	Medication review of medical notes
182884001	Pulse rate
78564009	Bowel cancer screening programme: faecal occult blood result
375211000000108	MCH - Mean corpuscular haemoglobin
368481000000103	Drug compliance good
1022471000000100	Seen by practice phlebotomist
93311000000106	Haemoglobin A1c level - International Federation of Clinical Chemistry and Laboratory Medicine standardised
373942005	Patient contact administration
185305000	Standing height
999791000000106	Blood pressure recorded by patient at home
401271004	Protocol entry
714241000000102	Red blood cell distribution width
413153004	Body mass index
394837003	Administrative reason for encounter
185351004	Seen in ophthalmology clinic
248333004	Letter encounter
60621009	Patient review
993501000000105	Telephone call to a patient
308720009	SMS text message sent to patient
185222001	E-mail sent to patient
763288003	Seen in orthopaedic clinic
24671000000101	White British - ethnic category 2001 census
494131000000105	NHS 111 report received
26079004	Serum calcium level
887641000000105	MCHC - Mean corpuscular haemoglobin concentration
304507003	Serum total bilirubin level
1000691000000100	Serum total protein
907151000000108	Serum cholesterol level
997591000000109	Letter received
1005671000000100	Serum triglycerides level

1022481000000100	Long term condition care planning invitation first letter
1000811000000100	Serum lipids level
198291000000105	Serum high density lipoprotein cholesterol level
912681000000107	Serum TSH (thyroid stimulating hormone) level
1005661000000100	Seen in dermatology clinic
1022791000000100	Brief history taken
1005681000000100	Administration
408363009	Patient understands why taking all medication
270422009	Serum low density lipoprotein cholesterol level
160240006	Informed consent given
225323000	Serum cholesterol/high density lipoprotein ratio
1015681000000100	Plan
1022191000000100	Enjoys light exercise
160631001	Lifestyle education
203031000000107	Moderate frailty
717371000000107	Serum adjusted calcium concentration
955691000000108	Seasonal influenza vaccination given by pharmacist
313204009	Mail administration procedure
935051000000108	Clinical letter
185276001	Seen in physiotherapy department
976631000000101	Serum folate level
416118004	Consent given for blood test
993381000000106	White: English or Welsh or Scottish or Northern Irish or British - England and Wales ethnic category 2011 census
993411000000108	Thyroid function test
59621000	Standard ECG
907131000000101	Urine nitrite negative
314138001	History/symptoms
308021002	Serum ferritin level
11429006	Consultation
1364181000000100	Direct-acting oral anticoagulant dose unchanged
164847006	Implied consent for core Summary Care Record dataset upload
1016851000000100	NHS Health Check completed
160237006	Blood test requested
307780009	Health education - exercise
167297006	Date
167261002	Urine blood test = negative
773011000000101	Urine glucose test negative

993391000000108	Did not attend
413672003	Serum vitamin B12 level
279039007	Liver function test
25781000000108	Urinalysis = no abnormality
523221000000100	Has shown no side effects from medication
1001231000000100	Awaiting clinical code migration to EMIS Web
49218002	Influenza vaccination invitation first short message service text message sent
314940005	Review of medication
777791000000102	Consent given for communication by text messaging
281399006	Serum globulin level
1572871000006100	Seen in ENT clinic
408508002	Seen in clinic
167221003	SARS-CoV-2 (severe acute respiratory syndrome coronavirus 2) vaccination invitation SMS (short message service) text message sent
1240781000000100	Audit administration
185223006	Seasonal influenza vaccination given by other healthcare provider
705025004	Consent given for communication by short message service text messaging
105542008	Mammography normal
250171008	Clinical history and observation findings
168749009	Consent given for communication by email
713481000000109	Percentage basophils
955651000000100	Out of hours report
165355002	Urine albumin:creatinine ratio
394700004	Face to face consultation
1015501000000100	Patient identity verified
439811001	Notes summary on computer
8392000	Urine protein test negative
736728004	Seen by ambulance crew
170557005	British or mixed British - ethnic category 2001 census
1023491000000100	Non-smoker
430193006	Chronic disease initial assessment
184229000	Patient informed - test result
306806004	Smoking cessation advice
167273002	Diet health education
183616001	Main spoken language English
185228002	Urine ketone test negative

719329004	High risk category for developing complication from COVID-19 infection
167287002	Admin. reminder
713776001	Urine creatinine level
1875551000006100	Medication review done by pharmacist
1007881000000100	Urine dipstick test
1300561000000100	Follow-up arranged
911361000000104	Consent given to receive test results by short message service text messaging
1003271000000100	Repeat medication check
763380007	Hypertension monitoring
315570003	GPPAQ hours in last week spent cycling - none
182838006	Alcohol intake
29857009	Seen in urology clinic
773051000000102	Express consent for core and additional Summary Care Record dataset upload
286261000000101	Seen in gastroenterology clinic
49727002	Serum non high density lipoprotein cholesterol level
185184005	Urine sample sent to Lab
185381007	12 lead ECG
1746291000006100	Seen in speech and language clinic
268400002	Peripheral oxygen saturation
431314004	Test result to patient by telephone
185220009	Diet education
275944005	Serum PSA (prostate specific antigen) level
165339009	Diet good
160573003	Letter sent to patient
185337004	Random sample
185293001	Magnetic resonance imaging
308447003	At risk of dementia
113091000	General practice physical activity questionnaire physical activity index: active
165357005	Implementation of aseptic technique
1127441000000100	Seen by consultant
840671000000108	Prescription
370822003	Medication review done by clinical pharmacist
366241000000106	Seen in cardiac clinic
260885003	GPPAQ not in employment
310500000	Failed encounter - no answer when rang back

50417007	Medicines reconciliation completed
14760008	Consent given to share patient data with specified third party
1746171000006100	Referral to social prescribing service
1000381000000100	Referral letter sent
698464007	Email sent to outside agency
46825001	Seen in GP's surgery
170625000	Inhaler technique - good
183093006	Mild frailty
185321005	Administration of medication under patient specific direction
928971000000108	Provision of chaperone refused
195967001	Letter encounter to patient
715931000000100	Discharged from hospital
709901000000106	Enjoys moderate exercise
319951000000105	Medication review without patient
185202000	Constipation
408562003	Urine microalbumin level
925791000000100	QOF (Quality and Outcomes Framework) quality indicator-related care invitation
160632008	Telephone call from a patient
413973005	Breast neoplasm screening normal
170638008	Urine leucocyte test = negative
526421000000109	Blood pressure monitoring invitation SMS (short message service) text message
183665006	Indication for each drug checked
1010251000000100	New medication commenced
35489007	Repeat prescription reviewed by pharmacist
968191000000100	Interpreter not needed
401176004	Patient contact details verified
1022511000000100	Seen in radiology department
1085181000000100	Neurology
394591006	Estimated creatinine clearance (Cockcroft-Gault formula)
408343002	Scanned document
811921000000103	Physical examination
1109921000000100	Long term condition care planning invitation second letter
903081000000107	Medication Reconciliation
266712008	Third party encounter
185181002	Seen by neurologist
183452005	Seen in general medical clinic

161891005	Informed consent given for treatment
315595002	Seen in out of hours centre
45326000	Drug side effects checked
912721000000100	NHS Health Check invitation short message service text message
5880005	Serum bicarbonate level
408947007	Discharge summary report
370208006	Marital state unknown
95324001	Hospital prescription
171024009	Medication changed
305684006	Advice about treatment given
1001371000000100	Serum chloride level
185318008	No new symptoms
762911000000102	Non - drinker
980641000000103	Patient's next of kin
1957991000006100	Influenza vaccination invitation first letter sent
170635006	Sampling of cervix for Papanicolaou smear done
24681000000104	Had a chat to patient
408356009	Emergency hospital admission
185241003	Referral letter sent by email
928451000000107	Serum CRP (C reactive protein) level
396275006	NHS Health Check invitation
200481000000107	Seen in musculoskeletal clinic
201101007	Dietary advice
1000681000000100	ESR - erythrocyte sedimentation rate
21522001	Able to use private transport
523241000000107	Lloyd George record received
394995008	Administration of medication under patient group direction
314503007	Post hospital discharge medication reconciliation with medical notes
928991000000107	Serum bilirubin level
1000671000000100	Alert received from telehealth monitoring system
30989003	Medication management
398838000	Administration of first dose of SARS-CoV-2 (severe acute respiratory syndrome coronavirus 2) vaccine
1746501000006100	Pulse regular
401270003	Health education - alcohol
711124002	Administration of second dose of SARS-CoV-2 (severe acute respiratory syndrome coronavirus 2) vaccine
427933004	GPPAQ usual level of walking pace - slow

999691000000104	Seen by clinical pharmacist
307541003	Initial memory assessment
182832007	No breathlessness
165791000000107	Patient advised about exercise
492811000000103	General practice physical activity questionnaire physical activity index: moderately inactive
1127431000000100	Blood test due
1324691000000100	ECG
404640003	Advice given
730061000000107	Reason for referral
14361000000109	Phlebotomy generated from secondary care done by practice
183073003	Infection screening
243790003	Bone profile
888901000000102	Provision of patient satisfaction questionnaire
271636001	Seen in mental health clinic
300627003	Seen in elderly care clinic
185719002	Serum free T4 level
1324681000000100	In-house physio
864231000000108	Referred for health coaching
699249000	Letter encounter from patient
335081000000105	Triage
1006761000000100	Additional note
418043000	Education about alcohol consumption
431957001	Temperature
77248004	Medication review done by pharmacy technician
185248009	Good compliance with inhaler
718087004	Wound care
716101000000104	Seen by physiotherapist
182878004	Repeat prescription monitoring
160629005	Medication review invitation
399208008	Current non-smoker
165333005	Home visit
276221000000100	Infection control procedure
719326006	Seen in colorectal clinic
270424005	GPPAQ hours in last week spent walking - 3 hours or more
167223000	General practice physical activity questionnaire physical activity index: inactive
1746271000006100	Referral for further care

4525004	General practice physical activity questionnaire physical activity index: moderately active
408373006	Medication increased
310065000	Referral to physiotherapist
390862006	X-ray report received
366979004	Advice to continue with drug treatment
855981000006102	Medication monitoring
281078001	Drug compliance checked
23056005	Telehealth monitoring
736601004	Non-diabetic hyperglycaemia
1000751000000100	Open access service
185285001	QRISK2 cardiovascular disease 10 year risk score
271739002	ECG: sinus rhythm
366121000000108	Renal profile
183444007	Seen in pain clinic
394671009	Demonstration of inhaler technique
37361000000105	Seen by occupational therapist
38341003	Phacoemulsification of lens and insertion of prosthetic replacement
2071000000108	Appointment made
225358003	Letter from Driver and Vehicle Licensing Authority received
703421000	Seen in general surgery clinic
170922004	GPPAQ hrs in last wk spent in physical exercise - 3hrs or more
295051000000101	Exercise physically impossible
401250009	Exercise education
426783006	Seen in accident and emergency department
1087551000000100	Short message service text message sent
440168007	Patient given telephone advice out of hours
700449008	Has authorisation for medication under PGD (patient group direction)
160618006	Provision of advice, assessment or treatment limited due to COVID-19 pandemic
711408005	Synchronisation of repeat medication
185287009	Advice to return if problem persists or deteriorates
313184003	Self-help advice leaflet given
1672851000006100	Invitation to participate in research study
172630005	Intramuscular injection of vitamin B12
185244006	10g monofilament sensation R foot normal
394675000	No response to bowel cancer screening programme invitation
439708006	Liquid based cervical cytology screening

1746371000006100	Opportunistic verification of patient mobile telephone number
395067002	White British
249274008	ECG normal
314373007	Smoking monitoring invitation
197480006	Wheeze absent
713056003	Discussion about clinical red flag warning sign
315236000	Depression screening using questions
810901000000102	Plasma glucose level
170614009	Optimisation of drug dosage
735259005	Drugs not issued
185731000	Failed encounter - short message service text message delivery failure
185229005	Reassurance given
394712000	Inhaler technique observed
927621000000101	No cough
822721000000100	GPPAQ hrs in last wk spent gardening/DIY-some but less than 1hr
1746461000006100	Email received from third party
1010671000000100	10g monofilament sensation L foot normal
310436000	Nucleated red blood cell count
417036008	Seen by Accident and Emergency service
77477000	CHA2DS2-VASc (congestive heart failure, hypertension, age 2, diabetes mellitus, stroke 2, vascular disease, age, sex category) stroke risk score
415693003	SMS text message received from patient
184156005	Email encounter from third party
82078001	Urine leucocyte test = +
134395001	360 degree sweep of cervix performed
183096003	Urinary symptoms
183039008	Provision of copy of letter from specialist to patient
1321171000000100	Has a carer
68566005	Dressing of wound
373251000000108	Incoming mail
394703002	Magnetic resonance imaging of spine
182850000	Vitamin D deficiency
515201000000103	O/E - Left posterior tibial pulse normal
1066951000000100	CT of head
182531007	Syringing ear to remove wax
32485007	Patient feels well
310429001	Outcome
185227007	Seen by Primary Care Navigator

394669009	Respiratory rate
162415008	O/E - right posterior tibial pulse normal
200971000000100	Consent given for minor surgery procedure
185307008	Fax sent to:
161898004	Cancer care review
720006006	O/E - right dorsalis pedis normal
1052411000000100	NHS Health Check verbal invitation
163133003	GPPAQ usual level of walking pace - steady
811611000000101	Abdomen examined - NAD
1746511000006100	Chest clear
34713006	Electronic record notes summary verified
414907000	Medical records review
1746431000006100	Neurological referral
10601006	GPPAQ hours in last week spent in physical exercise - none
802241000000106	Community health services
183631005	Blood sample -> Lab NOS
241645008	No known allergy
717661000000106	Seen in hospital out-pat.
170777000	Alcohol units consumed per week
267112005	Brief intervention for physical activity completed
198241000000100	Wax in ear
271921002	O/E - left dorsalis pedis normal
308722001	Smoking cessation education
14221000000108	Nocturia
134420004	Light drinker - 1-2u/day
185271006	No suicidal thoughts
15188001	Provision of patient questionnaire
871691000000100	Planned telephone contact
335661000000109	Complaining of a general symptom
522261000000101	Short message service text message received from patient
414912004	Self measured blood pressure reading
301708006	Social prescribing offered
160575005	Treatment plan given
148561000000109	Medicines adherence checked
314705003	GPPAQ hrs in last wk spent on house work/child care-3hrs or more
162409008	E-mail received from patient
413294000	Structured medication review

312853008	Colonoscopy
303653007	Advice given about weight management
168731009	ECG finding
1746241000006100	Medication review due
428489000	Inhaler technique not checked
86290005	Seen in breast clinic
1082641000000100	Signposting to GP (general practitioner)
1239511000000100	Falls
190634004	QOF (Quality and Outcomes Framework) non-diabetic hyperglycaemia quality indicator-related care invitation
716186003	Seen in respiratory clinic
314529007	Message given to patient
1084071000000100	English - ethnic category 2001 census
526431000000106	Referral to ear, nose and throat service
139394000	Clotting screening test
185211000	Seen in audiology clinic
414896001	Seen by general practitioner
81680005	Removal of skin sutures/clips
698471002	GPPAQ hours in last week spent gardening/DIY - none
267039000	Tobacco smoking behaviour - finding
162290004	GPPAQ hrs last wk spent house work/child care-1hr less than 3hrs
200491000000109	Influenza vaccination invitation second letter sent
1011481000000100	Prothrombin time
852471000000107	NHS Health Check invitation first letter
185190009	Teetotaller
254701007	Using inhaled steroids - normal dose
84229001	Chaperone present
16310003	Patient's condition improved
239873007	Bowel cancer screening programme

Supplementary Table 7. Codes used to search for each of the top 10 most common co-existing diagnoses before and after the confirmation of Parkinson’s disease.

Before the confirmation of PD diagnosis		
**Diagnosis**	**SnomedCTConceptId**	**Term**
**Musculoskeletal and connective tissue diseases**	81680005	Neck pain
	279039007	Low back pain
	45326000	Shoulder pain

	49218002	Hip pain
	30989003	Knee pain
	47933007	Foot pain
	239873007	Osteoarthritis of knee
	279069000	Musculoskeletal pain
	202794004	Lumbago with sciatica
	281245003	Musculoskeletal chest pain
	201819000	Generalised osteoarthritis
	23056005	Sciatica
	247366003	Acute back pain with sciatica
	57676002	Joint pain
	33952002	Localised osteoarthritis, unspecified, of the lower leg
	8847002	Osteoarthritis of spine
	161894002	C/O - low back pain
	396275006	Osteoarthritis NOS
	30989003	Knee joint pain
	427933004	Seen in musculoskeletal clinic
	239873007	Osteoarthritis NOS, of knee
	312225001	Musculoskeletal and connective tissue diseases
	239872002	Osteoarthritis of hip
	396275006	Osteoarthritis
	3723001	Arthritis
	312225001	Musculoskeletal and connective tissue disorder
	239733006	Anterior knee pain
	202482009	Wrist joint pain
	84869007	Musculoskeletal symptom
	413159000	Referral to musculoskeletal clinic
	279040009	Mechanical low back pain
	239872002	Osteoarthritis NOS, of hip
	396275006	Osteoarthritis and allied disorders
	275554004	[V]Personal history of arthritis
	161567008	H/O: rheumatoid arthritis
	202480001	Elbow joint pain
	450521003	Patellofemoral osteoarthritis
	202490009	Ankle joint pain

	396275006	Osteoarthritis NOS, of the lower leg
	268054009	Osteoarthritis of multiple joints
**Skin lesions & dermatitis**	162415008	C/O: a rash
	398838000	Seborrhoeic keratosis
	398838000	Seborrhoeic wart
	703938007	Inflammatory dermatosis
	43116000	Eczema
	201091002	Skin tag
	271807003	[D]Rash and other nonspecific skin eruption NOS
	442279002	Rash of genitalia
	24079001	Atopic dermatitis/eczema
	304386008	O/E - itchy rash
	201101007	Actinic keratosis
	254701007	Basal cell carcinoma of skin
	268911002	O/E - rash present
	57092006	Flexural eczema
	161561009	H/O: eczema
	40275004	Contact dermatitis and other eczemas
	80659006	Disorder of skin and/or subcutaneous tissue
	50563003	Seborrhoeic dermatitis
	366362000	Varicose eczema
	80659006	Disorder of skin AND/OR subcutaneous tissue
	24079001	Dermatitis/eczemas
	275700003	Varicose eczema - leg
	40275004	Contact dermatitis
	445111008	Cryotherapy of actinic keratosis
	162415008	Complaining of a rash
	24079001	Atopic dermatitis
**Hypertensive disorder**	59621000	Essential hypertension
	401118009	Hypertension annual review
	275944005	Hypertension monitoring
	401048005	Hypertension six month review
	38341003	Hypertensive disease
	185723005	Hypertension monitoring telephone invite

	38341003	BP - hypertensive disease
	185719002	Hypertension monitoring first letter
	185720008	Hypertension monitoring second letter
	308116003	Antihypertensive therapy
	170586008	Treatment for hypertension started
	38341003	Hypertensive disorder
	161501007	H/O: hypertension
	473225006	Hypertension medication review
	59621000	Essential hypertension NOS
	407567007	Patient on maximal tolerated antihypertensive therapy
	38341003	Hypertension
	185264001	Seen in hypertension clinic
**Headache & Migraine**	398057008	Tension headache
	25064002	Headache
	37796009	Migraine
	272027003	Complaining of a headache
	267096005	Frontal headache
	161481007	H/O: migraine
	272027003	C/O - a headache
	23186000	Menstrual migraine
	25064002	[D]Headache
	4969004	Sinus headache
	193030005	Migraine variants
**Depression & Anxiety**	413169006	On depression register
	197480006	Anxiety disorder
	231504006	Mixed anxiety and depressive disorder
	198288003	Anxiety state
	231504006	[X]Other mixed anxiety disorders
	35489007	Depression
	413973005	Depression interim review
	87414006	Reactive depression
	58703003	Postpartum depression
	161469008	H/O: depression
	35489007	Depressive disorder
	161470009	H/O: anxiety state

	207363009	Anxiety neurosis
	413974004	Depression medication review
	310495003	Mild depression
	268621008	Recurrent major depressive episodes
	87414006	Reactive depression (situational)
	191616006	Recurrent depression
	21897009	Generalised anxiety disorder
	310497006	Severe depression
	310496002	Moderate depression
**Haemorrhoids & Constipation**	70153002	Haemorrhoids
	70153002	Haemorrhoids NOS
	195453000	Internal haemorrhoids, simple
	70153002	Piles - haemorrhoids
	26373009	External thrombosed haemorrhoids
	14760008	Constipation NOS
	14760008	Constipation
	174359003	Haemorrhoids: ligate/excise
**Abdominal Hernia**	177860007	Primary repair of inguinal hernia
	84089009	Hiatus hernia
	396347007	Umbilical hernia
	84089009	Hiatal hernia
	413146008	Primary laparoscopic repair of inguinal hernia
	309752000	Hiatus hernia with gangrene
	39839004	Diaphragmatic hernia
	44946007	Repair of umbilical hernia
	396232000	Inguinal hernia
	73147001	Direct inguinal hernia
	236022004	Left inguinal hernia
	65626001	Indirect inguinal hernia
	236021006	Right inguinal hernia
	1171000119108	Unilateral recurrent inguinal hernia unspecified
	177862004	Primary mesh repair of inguinal hernia
	177860007	Primary repair of inguinal hernia NOS
**Respiratory tract infection**	50417007	Lower respiratory tract infection

	281794004	Viral upper respiratory tract infection
	195742007	Acute lower respiratory tract infection
	275498002	Respiratory tract infection
**Dyspepsia/Indigestion/Reflux**	162031009	Dyspepsia
	162031009	Indigestion symptoms
	266433003	Reflux oesophagitis
	162031009	Indigestion
	235595009	Gastro-oesophageal reflux
	225587003	Gastric reflux
	249511005	Flatulent dyspepsia
	266435005	Gastro-oesophageal reflux disease without oesophagitis
	698065002	Acid reflux
	414581006	Laryngopharyngeal reflux
	235595009	Gastroesophageal reflux disease
	266433003	Gastro-oesophageal reflux disease with oesophagitis
**UTI**	314940005	Suspected UTI (urinary tract infection)
	68566005	Urinary tract infection, site not specified
	197927001	Recurrent urinary tract infection
	68566005	Urinary tract infection
	68566005	Urinary tract infection, site not specified NOS
	68566005	UTI - Urinary tract infection

After the confirmation of PD diagnosis

Disease	SnomedCTConceptId	Term
**Musculoskeletal disorders**	23056005	Sciatica
	30989003	Knee pain
	279039007	Low back pain
	239872002	Osteoarthritis of hip
	134407002	Chronic back pain
	45326000	Shoulder pain
	396275006	Osteoarthritis
	3723001	Arthritis
	323291000119108	Osteoarthritis of left hip joint
	49218002	Hip pain

	161891005	Backache
	202794004	Lumbago with sciatica
	413159000	Referral to musculoskeletal clinic
	81680005	Neck pain
	239873007	Osteoarthritis of knee
	239874001	Osteoarthritis of ankle
	427933004	Seen in musculoskeletal clinic
	279069000	Musculoskeletal pain
	161894002	C/O - low back pain
	161891005	Back pain without radiation NOS
	69896004	Rheumatoid arthritis
	847261000000104	Rheumatoid arthritis annual review
	239792003	Seronegative rheumatoid arthritis
	230821000000102	Rehabilitation for musculoskeletal disorders
	279035001	Acute thoracic back pain
	312225001	Musculoskeletal and connective tissue diseases
	312225001	Musculoskeletal and connective tissue disorder
	373623009	Osteoarthritis of glenohumeral joint
	161891005	Back pain
	239872002	Osteoarthritis NOS, of hip
	161568003	H/O: osteoarthritis
	8847002	Osteoarthritis of spine
	841261000000105	Referral to community musculoskeletal service
	247366003	Acute back pain with sciatica
	1.07895E+15	Referral to musculoskeletal extended scope practitioner
	161894002	Complaining of low back pain
	371081002	Arthritis of knee
	239733006	Anterior knee pain
	374471000000101	Referral to musculoskeletal special interest general practitioner
	239863005	Osteoarthritis of spinal facet joint
	363215001	Musculoskeletal system physical examination
	279040009	Mechanical low back pain

**Hypertension**	59621000	Essential hypertension
	185720008	Hypertension monitoring second letter
	275944005	Hypertension monitoring
	185719002	Hypertension monitoring first letter
	401118009	Hypertension annual review
	185723005	Hypertension monitoring telephone invite
	1.06695E+15	Hypertension monitoring SMS (short message service) text message first invitation
	38341003	Hypertensive disorder
	185264001	Seen in hypertension clinic
	1.06696E+15	Hypertension monitoring SMS (short message service) text message second invitation
	38341003	Hypertension
	1.06694E+15	Hypertension monitoring invitation SMS (short message service) text message
	407567007	Patient on maximal tolerated antihypertensive therapy
	185721007	Hypertension monitoring third letter
	170577003	Good hypertension control
	161501007	H/O: hypertension
	310427004	Hypertension monitoring invitation
	38341003	Hypertensive disease
	401048005	Hypertension six month review
	417312002	Suspected hypertension
**Urinary tract infection**	307541003	Lower urinary tract symptoms
	314940005	Suspected UTI (urinary tract infection)
	4009004	UTI - Lower urinary tract infection
	68566005	UTI - Urinary tract infection
	197927001	Recurrent urinary tract infection
	68566005	Urinary tract infection
	68566005	Urinary tract infection, site not specified
	721104000	Sepsis due to urinary tract infection
	197927001	Recurrent urinary tract infections

	307541003	LUTS - Lower urinary tract symptoms
**Skin lesions**	398838000	Seborrhoeic keratosis
	398838000	Senile hyperkeratosis
	95324001	Skin lesion
	201101007	Actinic keratosis
	201101007	Solar keratosis
	254666005	Keratosis
	315260001	Subcutaneous component of pigmented skin lesion
	315258003	Crusting of pigmented skin lesion
**Diabetes**	170777000	Diabetic annual review
	394675000	O/E - Left diabetic foot at low risk
	170742000	Diabetic monitoring
	394671009	O/E - Right diabetic foot at low risk
	185229005	Seen in diabetic clinic
	390850007	O/E - no right diabetic retinopathy
	170745003	Diabetic on diet only
	754121000000107	Type II diabetic dietary review
	44054006	Type 2 diabetes mellitus
	134395001	Diabetic retinopathy screening
	811981000000102	Diabetes self-management plan agreed
	313340009	Seen in diabetic eye clinic
	170746002	Diabetic on oral treatment
	887861000000105	Diabetes Year of Care annual review
	170744004	Follow-up diabetic assessment
	4855003	Diabetic retinopathy
	390834004	Background diabetic retinopathy
	170752001	Has seen dietician - diabetes
	390853009	O/E - no left diabetic retinopathy
	415270003	Referral to diabetes structured education programme
	1.06691E+15	Diabetes monitoring SMS (short message service) text message first invitation
	700449008	Non-diabetic hyperglycaemia
	170763003	Diabetic - good control

	754081000000109	Diabetic dietary review
	394682001	O/E - Right diabetic foot at moderate risk
	700414001	Education about diabetes and driving
	185756006	Diabetes monitoring first letter
	284350006	Diabetes mellitus diet education
	705072004	Diabetes monitoring invitation by short message service text messaging
	408409007	O/E - right eye background diabetic retinopathy
	185757002	Diabetes monitoring second letter
	394681008	O/E - Left diabetic foot at moderate risk
	73211009	Diabetes mellitus
	703040004	Agreeing on diabetes care plan
	314194001	Diabetic on insulin and oral treatment
	755491000000100	Diabetes structured education programme completed
	170742000	Diabetes management plan given
	394725008	Diabetes medication review
	923461000000103	Lifestyle education for diabetes
	248161000000101	Referral for diabetic retinopathy screening
	185760009	Diabetes monitoring telephone invite
	407672007	Seen in diabetic foot clinic
**Depression & Anxiety**	198288003	Anxiety state
	191616006	Recurrent depression
	413973005	Depression interim review
	1.83956E+15	PHQ9 total score 10-14 (moderate depression)
	197480006	Anxiety disorder
	231504006	Mixed anxiety and depressive disorder
	413974004	Depression medication review
	35489007	Depression
	197480006	[X]Anxiety NOS
	35489007	Depressive disorder

	70997004	Mild anxiety
	413972000	Depression annual review
	21897009	Generalised anxiety disorder
	445455005	Generalized Anxiety Disorder 7 item score
	48694002	Anxiety
	394924000	Symptoms of depression
**Haemorrhoids & Constipation**	70153002	Haemorrhoids
	14760008	Constipation
	14760008	Constipation NOS
	31499008	Chronic constipation with overflow
	70153002	Piles - haemorrhoids
	236069009	Chronic constipation
**Asthma**	394700004	Asthma annual review
	195967001	Asthma
	170635006	Asthma not disturbing sleep
	928451000000107	Asthma monitoring invitation SMS (short message service) text message
	527171000000103	Patient has a written asthma personal action plan
	170638008	Asthma not limiting activities
	406162001	Asthma management
	370208006	Asthma never causes daytime symptoms
	185731000	Asthma monitoring call first letter
	2.01003E+15	Acute infective exacerbation of asthma
	811921000000103	Asthma self-management plan agreed
	401182001	Asthma monitoring by nurse
	185732007	Asthma monitoring call second letter
	394720003	Asthma medication review
	394701000	Asthma follow-up
	340911000000109	Asthma trigger - pollen
	394967008	Suspected asthma
	370202007	Asthma causes daytime symptoms 1 to 2 times per month

	810901000000102	Asthma self-management plan review
	161527007	H/O: asthma
	754061000000100	Asthma review using Royal College of Physicians three questions
	959401000000101	Asthma monitoring SMS (short message service) text message first invitation
	370218001	Mild asthma
	270442000	Asthma monitoring check done
	170636007	Asthma never disturbs sleep
	170658009	Asthma never restricts exercise
	401135008	Health education - asthma
	195967001	Bronchial asthma
	170656008	Asthma sometimes restricts exercise
	771901000000100	Asthma causes night time symptoms 1 to 2 times per week
	892301000000100	Asthma management plan declined
	185734008	Asthma monitoring call third letter
	31387002	Exercise-induced asthma
	370203002	Asthma causes daytime symptoms 1 to 2 times per week
	170634005	Asthma disturbs sleep frequently
	182729000	Asthma control step 3
	401183006	Asthma monitoring by doctor
	370204008	Asthma causes daytime symptoms most days
	340901000000107	Asthma trigger - exercise
	170632009	Asthma causing night waking
	170657004	Asthma severely restricts exercise
	373899003	Asthma daytime symptoms
	866881000000101	Chronic asthma with fixed airflow obstruction
	708038006	Acute exacerbation of asthma
	195967001	Asthma NOS
	185728001	Attends asthma monitoring
	233683003	Pollen asthma
	182726007	Asthma control step 0
	182728008	Asthma control step 2

	182727003	Asthma control step 1
	370207001	Asthma limits walking up hills or stairs
	772051000000106	Asthma limits activities most days
	185730004	Asthma monitor offer default
	390877003	Step up change in asthma management plan
	370205009	Asthma causes night symptoms 1 to 2 times per month
	867171000000106	No asthma trigger identified by subject
	708373002	Emergency asthma patient visit since last encounter
	473391009	Asthma never causes night symptoms
	201051000000101	Asthma trigger: animals
	312453004	Asthma - currently active
	959421000000105	Asthma monitoring SMS (short message service) text message second invitation
	713701000000108	Asthma monitoring administration
**Respiratory tract infection**	50417007	Lower respiratory tract infection
	281794004	Viral upper respiratory tract infection
	275498002	Respiratory tract infection
	195742007	Acute lower respiratory tract infection
**Rheumatoid arthritis**	185227007	Seen in rheumatology clinic
	183526002	Referred to rheumatologist
	69896004	Rheumatoid arthritis
	847261000000104	Rheumatoid arthritis annual review
	239792003	Seronegative rheumatoid arthritis
	401192009	Disease modifying antirheumatic drug monitoring
	364801000000104	Seen in general practitioner disease modifying antirheumatic drug monitoring clinic
	305749000	Seen by rheumatology nurse specialist
	306290009	Referral to rheumatologist
