# Supplementary material for: AccessPD as a next generation registry to accelerate Parkinson’s disease research
Source: NPJ Parkinsons Dis. 2024 Mar 19;10:66. doi: 10.1038/s41531-024-00651-z (PMC10950855; doi:10.1038/s41531-024-00651-z)
Supplement: Supplementary file 2 — Reporting summary [file 41531_2024_651_MOESM2_ESM.pdf]

nature portfolio

Corresponding author(s): Prof Alastair Noyce

Last updated by author(s): 2023/11/28

Reporting Summary

Nature Portfolio wishes to improve the reproducibility of the work that we publish. This form provides structure for consistency and transparency in reporting. For further information on Nature Portfolio policies, see our Editorial Policies and the Editorial Policy Checklist.

Statistics

For all statistical analyses, confirm that the following items are present in the figure legend, table legend, main text, or Methods section.

n/a	Confirmed	
☐	☒	The exact sample size (*n*) for each experimental group/condition, given as a discrete number and unit of measurement
☐	☒	A statement on whether measurements were taken from distinct samples or whether the same sample was measured repeatedly
☒	☐	The statistical test(s) used AND whether they are one- or two-sided<br>*Only common tests should be described solely by name; describe more complex techniques in the Methods section.*
☒	☐	A description of all covariates tested
☒	☐	A description of any assumptions or corrections, such as tests of normality and adjustment for multiple comparisons
☐	☒	A full description of the statistical parameters including central tendency (e.g. means) or other basic estimates (e.g. regression coefficient) AND variation (e.g. standard deviation) or associated estimates of uncertainty (e.g. confidence intervals)
☒	☐	For null hypothesis testing, the test statistic (e.g. *F*, *t*, *r*) with confidence intervals, effect sizes, degrees of freedom and *P* value noted<br>*Give P values as exact values whenever suitable.*
☒	☐	For Bayesian analysis, information on the choice of priors and Markov chain Monte Carlo settings
☒	☐	For hierarchical and complex designs, identification of the appropriate level for tests and full reporting of outcomes
☒	☐	Estimates of effect sizes (e.g. Cohen's *d*, Pearson's *r*), indicating how they were calculated

Our web collection on statistics for biologists contains articles on many of the points above.

Software and code

Policy information about availability of computer code

Data collection	No software was used.
Data analysis	Python v3 and Jupyter notebooks were used for data analysis

For manuscripts utilizing custom algorithms or software that are central to the research but not yet described in published literature, software must be made available to editors and reviewers. We strongly encourage code deposition in a community repository (e.g. GitHub). See the Nature Portfolio guidelines for submitting code & software for further information.

Data

Policy information about availability of data

All manuscripts must include a data availability statement. This statement should provide the following information, where applicable:

- Accession codes, unique identifiers, or web links for publicly available datasets
- A description of any restrictions on data availability
- For clinical datasets or third party data, please ensure that the statement adheres to our policy

The datasets generated and/or analyzed during the current study are not publicly available due to privacy restrictions but are available from the corresponding author on reasonable request.

Research involving human participants, their data, or biological material

Policy information about studies with human participants or human data. See also policy information about sex, gender (identity/presentation), and sexual orientation and race, ethnicity and racism.

Reporting on sex and gender	Primary care EHR data of the consented participants were incorporated into the registry to provide a longitudinal view of the medical history, including gender. Of the 200 consented participants, 43.0% were female and 57.0% were male.
Reporting on race, ethnicity, or other socially relevant groupings	When asked about their ethnicity, the majority (94.5%) of the participants identified as white (including English, Welsh, Scottish, Northern Irish or Irish and any other White background), with the remainder disclosing that they were Asian, mixed, black or preferred not to answer. This categorization of ethnicities follows the ethnicity groups defined by the 2021 Census of England and Wales.
Population characteristics	The average age at the time of recruitment was 70.8 years. The youngest participant was 39-year-old and the oldest was 90-year-old. 18 (9.0%) participants were diagnosed before the age of 50. With an average number of years since diagnosis of 5.2 years, the participant with the longest history of PD was diagnosed 31 years ago, and 27.5% of participants were diagnosed within the past 24 months.
Recruitment	AccessPD recruits patients via a digital platform powered by uMedeor LTD (uMed), a life sciences research organization that acts as a data processor for a network of primary care general practitioners (GPs) across England. Patients with a coded diagnosis of PD – SNOMED-CT code 49049000 in primary care EHR – were identified as potential participants. With approval from participating GPs to engage these patients, uMed sends out a letter to inform them of the study opportunity, followed by a text message (either SMS or Email message) with an individualized link to the study portal. Once eligibility is established, participants give their consent to join the registry by going through a series of interactive questions. Alternatively, those less familiar with remote studies can request assistance from a study nurse who will go through the consent questions on the telephone with them and record answers in the database.
Ethics oversight	The study protocol was approved by East Midlands - Derby Research Ethics Committee in May 2022.

Note that full information on the approval of the study protocol must also be provided in the manuscript.

Field-specific reporting

Please select the one below that is the best fit for your research. If you are not sure, read the appropriate sections before making your selection.

☒ Life sciences ☐ Behavioural & social sciences ☐ Ecological, evolutionary & environmental sciences

For a reference copy of the document with all sections, see nature.com/documents/nr-reporting-summary-flat.pdf

Life sciences study design

All studies must disclose on these points even when the disclosure is negative.

Sample size	200 consented and agreed to take part in AccessPD.
Data exclusions	We excluded 1 participant from the analysis because they reported having a multiple system atrophy (MSA) diagnosis.
Replication	No replication analysis was performed.
Randomization	This is not relevant to our study design as the objective of the study is to create a large-scale database.
Blinding	Blinding was not relevant to our study because this is a registry instead of a clinical trial.

Reporting for specific materials, systems and methods

We require information from authors about some types of materials, experimental systems and methods used in many studies. Here, indicate whether each material, system or method listed is relevant to your study. If you are not sure if a list item applies to your research, read the appropriate section before selecting a response.

Materials & experimental systems

n/a	Involved in the study
☒	☐ Antibodies
☒	☐ Eukaryotic cell lines
☒	☐ Palaeontology and archaeology
☒	☐ Animals and other organisms
☐	☒ Clinical data
☒	☐ Dual use research of concern
☒	☐ Plants

Methods

n/a	Involved in the study
☒	☐ ChIP-seq
☒	☐ Flow cytometry
☒	☐ MRI-based neuroimaging

Clinical data

Policy information about clinical studies

All manuscripts should comply with the ICMJE guidelines for publication of clinical research and a completed CONSORT checklist must be included with all submissions.

Clinical trial registration: N/A

Study protocol: N/A

Data collection: It is an ongoing registry in England. Patients are recruited from primary care providers in England.

Outcomes: It is a disease registry.

Plants

Seed stocks: *Report on the source of all seed stocks or other plant material used. If applicable, state the seed stock centre and catalogue number. If plant specimens were collected from the field, describe the collection location, date and sampling procedures.*

Novel plant genotypes: *Describe the methods by which all novel plant genotypes were produced. This includes those generated by transgenic approaches, gene editing, chemical/radiation-based mutagenesis and hybridization. For transgenic lines, describe the transformation method, the number of independent lines analyzed and the generation upon which experiments were performed. For gene-edited lines, describe the editor used, the endogenous sequence targeted for editing, the targeting guide RNA sequence (if applicable) and how the editor was applied.*

Authentication: *Describe any authentication procedures for each seed stock used or novel genotype generated. Describe any experiments used to assess the effect of a mutation and, where applicable, how potential secondary effects (e.g. second site T-DNA insertions, mosiacism, off-target gene editing) were examined.*
